# uL3 Regulates Redox Metabolism and Ferroptosis Sensitivity of p53-Deleted Colorectal Cancer Cells

**DOI:** 10.3390/antiox13070757

**Published:** 2024-06-22

**Authors:** Chiara Brignola, Annalisa Pecoraro, Camilla Danisi, Nunzia Iaccarino, Anna Di Porzio, Francesca Romano, Pietro Carotenuto, Giulia Russo, Annapina Russo

**Affiliations:** 1 Department of Pharmacy, University of Naples “Federico II”, Via Domenico Montesano, 49, 80131 Naples, Italy; chiara.brignola@unina.it (C.B.); annalisa.pecoraro@unina.it (A.P.); camilla.danisi@unina.it (C.D.); nunzia.iaccarino@unina.it (N.I.); anna.diporzio@unina.it (A.D.P.); francesca.romano2@unina.it (F.R.); giulia.russo@unina.it (G.R.); 2 TIGEM, Telethon Institute of Genetics and Medicine, Via Campi Flegrei, 34, 80078 Naples, Italy; p.carotenuto@tigem.it; 3 Medical Genetics, Department of Translational Medical Science, University of Naples “Federico II”, Corso Umberto I, 40, 80138 Naples, Italy

**Keywords:** drug resistance, cancer redox metabolic reprogramming, SLC7A11/xCT, ferroptosis, ribosomal protein uL3, CAM assay

## Abstract

Despite advancements in therapeutic strategies, the development of drug resistance and metastasis remains a serious concern for the efficacy of chemotherapy against colorectal cancer (CRC). We have previously demonstrated that low expression of ribosomal protein uL3 positively correlates with chemoresistance in CRC patients. Here, we demonstrated that the loss of uL3 increased the metastatic capacity of CRC cells in chick embryos. Metabolomic analysis revealed large perturbations in amino acid and glutathione metabolism in resistant uL3-silenced CRC cells, indicating that uL3 silencing dramatically triggered redox metabolic reprogramming. RNA-Seq data revealed a notable dysregulation of 108 genes related to ferroptosis in CRC patients. Solute Carrier Family 7 Member 11 (SLC7A11) is one of the most dysregulated genes; its mRNA stability is negatively regulated by uL3, and its expression is inversely correlated with uL3 levels. Inhibition of SLC7A11 with erastin impaired resistant uL3-silenced CRC cell survival by inducing ferroptosis. Of interest, the combined treatment erastin plus uL3 enhanced the chemotherapeutic sensitivity of uL3-silenced CRC cells to erastin. The antimetastatic potential of the combined strategy was evaluated in chick embryos. Overall, our study sheds light on uL3-mediated chemoresistance and provides evidence of a novel therapeutic approach, erastin plus uL3, to induce ferroptosis, establishing individualized therapy by examining p53, uL3 and SLC7A11 profiles in tumors.

## 1. Introduction

Colorectal cancer (CRC) is the most common malignant tumor of the lower digestive tract around the world, with high morbidity and fatality. Nowadays, the treatment of CRC usually includes surgery, targeted therapy, radiotherapy, and chemotherapy [1]. Chemotherapy can be applied at different stages of the treatment and is commonly provided after surgery as adjuvant therapy for patients with advanced CRC. However, it remains a palliative therapy since most CRC patients ultimately develop drug resistance. In fact, chemotherapy failure can be observed in more than 90% of patients with metastatic cancer due to drug resistance [2]. Thus, how to overcome drug resistance remains one of the major challenges in this field.

Several studies indicate that many ribosomal proteins, in addition to their role as components of translational machinery, exert a variety of extra-ribosomal functions [3,4,5]. Evidence has emerged in recent decades regarding the close link between dysregulation in ribosomal proteins and the development of drug resistance in cancer cells that contain mutant p53 or no p53 at all [6,7]. We have previously demonstrated that the downregulation of ribosomal protein uL3 (formerly rpL3) positively correlates with drug resistance in CRC and lung cancer cells lacking active p53, and uL3 status is essential for cell response to anticancer drugs such as 5-fluorouracil (5-FU), oxaliplatin, and actinomycin D (Act D). The reduction of uL3 expression levels is associated with increased cell migration and proliferation, apoptosis inhibition, autophagy enhancement, and alteration of the epithelial–mesenchymal transition (EMT) program [8,9]. In addition, more recently, transcriptome analysis of a large cohort of CRC patients unveiled a strict correlation between uL3 expression and CRC patients’ outcomes. Specifically, the reduced uL3 levels were associated with a poor response to therapeutic treatment and higher expression of ATP-binding cassette (ABC) proteins, which actively expel the drug from the cells [10]. These studies have identified uL3 as an important player in response to chemotherapeutic drugs, suggesting a possible application of uL3 as a predictive biomarker of treatment response in CRC. In addition, we have previously demonstrated that the restoration of uL3 could be a strategy to resensitize 5-FU-resistant tumors lacking p53 and uL3 [11]. 

It is well established that oncogene activation, elevated metabolic activity, and compromised mitochondrial function cause cancer cells to undergo higher levels of oxidative stress than normal cells [12]. In order to resist the deadly consequences of oxidative stress, cancer cells need to establish defense mechanisms. Numerous studies point to metabolic reprogramming as one of these mechanisms facilitating tumor proliferation and invasion, as well as the development of a drug-resistant phenotype [13,14]. Metabolic adaptations in cancer cells may include dysregulated catabolism/anabolism of fatty acids and amino acids (AAs) [15]. Drug-resistant cells can alter AA metabolism to increase the production of reduced glutathione (GSH) and subsequently enhance their antioxidant defense [16]. A crucial modulator of cellular redox status is the cystine/glutamate antiporter system xc^−^ composed of the twelve-pass transmembrane transporter protein Solute Carrier Family 7 Member 11 (SLC7A11, also known as xCT), and a single-pass transmembrane regulator protein, SLC3A2. SLC7A11 allows the import of cystine, which is a rate-limiting phase in the production of GSH [17].

GSH content and redox status are involved in the regulation of ferroptosis, a non-apoptotic and iron-dependent form of regulated cell death that is characterized by the oxidization of polyunsaturated fatty acids and the accumulation of excessive lipid peroxides and reactive oxygen species (ROS) [18]. Ferroptosis has been studied in many human diseases, including cancer. Activation of ferroptosis has been demonstrated to inhibit the proliferation of malignant cells in a range of tumors, such as liver, breast, prostate, and pancreatic cancers [19,20]. Moreover, it has been shown that some highly aggressive cancer cells are inherently vulnerable to ferroptosis; as a result, inducing ferroptosis may become a potential cancer therapeutic strategy. Several compounds have been reported to act as ferroptosis inducers, including erastin, the first compound reported to trigger ferroptosis [21]. 

In this paper, we aimed to shed light on specific molecular signatures associated with uL3-based drug resistance that can be relevant in identifying vulnerability to specific programmed cell death. For this purpose, we performed metabolomic and transcriptomic analyses of CRC cells expressing uL3 or not, providing a detailed cancer profile of these cells. Analysis of differential patterns of genes and metabolites revealed that uL3 silencing mainly alters AA metabolism and GSH biosynthesis and generates a targetable vulnerability to ferroptosis. We have previously demonstrated that uL3 acts as a transcriptional repressor of *SLC7A11* gene in p53-mutated lung cancer cells [11]. Here, we reported the ability of uL3 to negatively regulate the mRNA stability of *SLC7A11* and demonstrated that pharmacological inhibition of SLC7A11 by erastin caused cell death in chemoresistant uL3-silenced CRC cells by activation of ferroptosis. Further, the knowledge of uL3 as a negative regulator of SLC7A11 prompted us to hypothesize that uL3 may have significant value in terms of the efficacy of erastin-based cytotoxicity. Of interest, the combined treatment with erastin plus uL3 was more effective than erastin alone in resistant uL3-silenced CRC cells. The effects of the combined treatment on tumor growth and invasiveness were analyzed in uL3-silenced CRC cell-derived Chorioallantoic Membrane (CAM) xenografts. Overall, our results demonstrated that SLC7A11 blocking by using erastin plus uL3 represents a promising option for tumors expressing low levels of uL3 and p53 and high levels of SLC7A11, and that are resistant to chemotherapeutic drugs.

## 2. Materials and Methods

### 2.1. Materials

Dulbecco’s Modified Eagle Medium (DMEM), Fetal Bovine Serum (FBS), L-Glutamine, Penicillin-Streptomycin and Phosphate-Buffered Saline (PBS) were obtained from Gibco™ (Grand Island, NE, USA). Matrigel^®^ was purchased from Corning^®^ (Corning, NY, USA). Lipofectamine™ 3000 was obtained from Invitrogen (Waltham, MA, USA). SLC7A11 siRNA and a negative control siRNA were synthesized by Integrated DNA Technologies, Inc. (Leuven, Belgium). TriFast™ was purchased from Euroclone S.p.A. (Milan, Italy). SensiFAST cDNA Synthesis kit (BIO-65054) and SensiFAST SYBER No-ROX kit (BIO-98020) were purchased from Meridian Bioscience, Inc. (Cincinnati, OH, USA). Proteinase K, 5-FU, Act D, Cycloheximide (CHX), cOmplete™ Mini, EDTA-free Protease Inhibitor Cocktail, 3-(4,5-dimethylthiazol-2-yl)-2,5-diphenyl tetrazolium bromide (MTT), RNase, propidium iodide (PI), 5-Sulfosalicylic acid (SSA), and Iron Assay Kit (MAK025) were purchased from Merck KGaA (Darmstadt, Germany). Ethanol and methanol were purchased from Thermo Fisher Scientific (Waltham, MA, USA). TruSeq™ RNA Sample Preparation Kit and TruSeq™ PE Cluster Kit v3 were purchased from Illumina, Inc. (San Diego, CA, USA). High Sensitivity DNA Assay Kit was purchased from Agilent Technologies, Inc. (La Jolla, CA, USA). Erastin (S7242) and Ferrostatin-1 (S7243) were obtained from Selleck Chemicals (Cologne, Germany). Z-VAD-FMK (219007) was obtained from EMD Millipore Corporation (Darmstadt, Germany). Necrosulfonamide (5025) was purchased from Tocris Bioscience (Bristol, UK). Tali™ Apoptosis Kit (A10788) was purchased from Life Technologies (Carlsbad, CA, USA). GSSG/GSH Quantification Kit (G257) and Cystine Uptake Assay Kit (UP05) were obtained from Dojindo Laboratories (Kumamoto, Japan). OxiSelect™ Intracellular ROS Assay Kit (STA-392) was obtained from Cell Biolabs, Inc. (San Diego, CA, USA). The antibodies to Nrf2 (sc-13032) and HA-probe (F-7) (sc-7392) were purchased from Santa Cruz (Dallas, TX, USA). Anti-SLC7A11 (12691), Anti-p21 (37543), and Anti-GAPDH (2128) were obtained from Cell Signaling Technology (Danvers, MA, USA). Anti-TfR1 (A85952) was obtained from Antibodies.com (Stockholm, Sweden). Anti-GPX4 (E-AB-64550), Anti-α-tubulin (E-AB-20036) and Excellent Chemiluminescent Substrate (ECL) Detection Kit (E-IR-R301) were purchased from Elabscience^®^ (Houston, TX, USA). 

All buffers and solutions were prepared with ultra-high-quality water. All reagents were of the purest commercial grade.

### 2.2. Cell Cultures and Transfection

HCT 116^p53−/−^ cells (American Type Culture Collection, (ATCC) Manassas, VA, USA) and uL3ΔHCT 116^p53−/−^, a cell line derived from HCT 116^p53−/−^ cells and stably silenced of uL3 [8], were cultured in DMEM, supplemented with 10% FBS, 2 mM L-Glutamine and 50 U/mL Penicillin-Streptomycin, under a humidified atmosphere of 5% CO_2_ at 37 °C.

Transfection of the plasmid encoding recombinant HA-uL3 (pHA-uL3), an empty vector plasmid, *SLC7A11* siRNA or the negative control siRNA was performed in cells using Lipofectamine™ 3000 according to the manufacturer’s instructions.

### 2.3. In Ovo CAM Assay

The CAM assay was performed on fertilized chick eggs, which were incubated in a rotating incubator at 37.8 °C and at a relative humidity of 50%. The first day of incubation was considered as egg development day (EDD) zero (EDD0). On EDD4, the eggs were opened: firstly, a small hole was punched into the eggshell at the “bottom” of the egg; then, around 3 mL of albumen was removed using a syringe, and the hole was closed with adhesive tape; subsequently, a window was cut on the top side of the egg using sharp scissors; finally, the window was closed with an additional strip of tape to prevent drying out and contaminating the CAM. The opened eggs were further kept in a stationary incubator. On EDD9, 20 µL of the suspension of CRC cells (3 × 10^6^ cells) in serum-free medium mixed with 20 µL of Matrigel was applied close to the allantoid vein bifurcation using a pipette while avoiding direct contact with the CAM. After xenografting, the eggs were left standing upright for 5–10 min in order to allow the cells to settle and then sealed with adhesive tape. The eggs were subsequently placed back in the incubator and kept until EDD14 or EDD17. During this period, frequent inspections through the window in the eggshell were performed to evaluate tumor growth and to confirm that the chick embryos were still alive. Tumor sizes were measured by excising and recording their wet weight on EDD14 or EDD17. The tumor volume was measured with a digital caliper and calculated using the ellipsoid formula (length × width × height × 0.52) in mm^3^. In the European Union countries, experimentation with chick embryos is allowed and does not require ethical approval from animal experimentation committees, on the condition that experiments begin and end before hatching.

### 2.4. Isolation of Organs from Chick Embryo

The embryos were dissected on EDD14 or EDD17 to isolate liver and lungs. Briefly, the embryo was decanted from the cut shell and transferred to a clean Petri dish breast side up. Each isolated embryo was washed several times with PBS and transferred to another fresh Petri dish containing PBS. The embryo was dissected by cutting through the sternum, and the internal organs were subsequently removed. To isolate the lungs, the rib cage was opened, the breast plate was separated, and the heart was excised. Finally, harvested tissue samples were frozen in liquid nitrogen and stored at −80 °C until further processing.

### 2.5. Quantitative Reverse Transcription Polymerase Chain Reaction (RT-qPCR)

Total RNA was extracted using TriFast™ following the manufacturer’s instructions. RNA was retrotranscribed using SensiFAST cDNA Synthesis kit, and then quantitative PCR was carried out using SensiFAST SYBER No-ROX kit. The primers are indicated in Table 1. The comparative Ct method was used to calculate the relative abundance of the mRNA and compared with that of β-actin expression [22].

### 2.6. Genomic DNA Isolation and Analysis of Human-Specific Alu Sequences by qPCR

For dissemination detection using human-specific quantitative Alu-PCR, embryo livers and lungs were thawed and processed using a digestion buffer containing 60 mM Tris pH 8.0, 100 mM EDTA, 0.5% SDS, and 500 µg/mL Proteinase K. Cell lysis was obtained by incubating the samples at 55 °C for approximately 16 h. Genomic DNA (gDNA) preparation was performed with phenol:chloroform extraction twice; then, the aqueous phase was placed in a fresh 2 mL tube, and 1/10 volume 3 M sodium acetate (pH 5.2) and 1 volume 95% ethanol at RT were added. gDNA was resuspended in an appropriate volume of TE, pH 7.4. Quantification and purity of isolated gDNA were determined using a NanoDrop™ Lite Spectrophotometer (Thermo Fisher Scientific, Waltham, MA, USA). Then, 30 ng of total gDNA was amplified by the application of the SensiFAST SYBER No-ROX kit and specific primers for human Alu (h-*Alu*) sequences (Table 2). gDNA samples were subjected to an initial denaturation at 95 °C for 15 min, followed by 40 cycles at 94 °C for 15 s for further denaturation, 60 °C for 30 s for annealing, and 72 °C for 30 s for elongation. The number of h-*Alu* sequences was normalized to chick GAPDH (ch-*GAPDH*), which was detected using the primers indicated in Table 2.

gDNA extracted from HCT 116^p53−/−^ or uL3ΔHCT 116^p53−/−^ cells was used to generate a standard curve using a dilution series (10^2^, 10^3^, and 10^4^). The standard curve was subsequently utilized to quantify human tumor cells in chick embryo tissues through Ct values obtained in triplicate. The specificity of qPCR reactions was verified by melting curve analysis.

### 2.7. Metabolite Extraction for NMR Analysis

HCT 116^p53−/−^ and uL3ΔHCT 116^p53−/−^ cells were seeded into 100 mm tissue culture plates, cultured for the night, and then treated with 5-FU (10 μM) for 48 h. After treatment, the culture medium was removed, and the cells were processed for the endo-metabolomic analysis. In brief, the cells were extensively washed (2 times) with warm PBS to completely remove any residue of culture medium. Then, PBS was removed, and ice-cold methanol (3 + 1 mL) was added to the cell plates to both quench the metabolism and disrupt the cell membrane, causing extraction of metabolites. The methanol extracts were then collected in 15 mL Falcon tubes and immersed into liquid nitrogen upon complete freezing. Samples were stored at −80 °C until NMR analysis.

### 2.8. NMR Spectroscopy

Frozen extracts were thawed at room temperature and transferred into glass vials. Subsequently, the samples were dried using a SpeedVac Concentrator (Thermo Fisher Scientific, Waltham, MA, USA) for 4 h and then lyophilized overnight to remove residual solvents. The dried samples were dissolved in 630 µL of sodium phosphate buffer (pH 7.4) and 70 µL of D_2_O (9:1 volume ratio), vortexed, and carefully transferred into Eppendorf tubes. The samples were then centrifuged at 15,000 rpm for 15 min at 4 °C before being transferred into 5 mm NMR tubes for analysis.

One-dimensional ^1^H-NMR spectra were recorded at 298 K using a Bruker Avance NEO 600 MHz spectrometer (Bruker Biospin GmbH, Rheinstetten, Germany) equipped with a QCI cryo-probe for 5 mm sample tubes and an autosampler SampleJet™ (Bruker Biospin GmbH, Rheinstetten, Germany). The spectra of hydrophilic cell extracts were obtained using Topspin 4.1 software (Bruker Biospin GmbH, Rheinstetten, Germany) and the ‘noesygppr1d’ pulse sequence, which allows for quantitative evaluation close to the water signal, pre-saturated at 4.698 ppm. Samples were held at 298 K for 300 s in the probe before beginning the experiment. The acquisition parameters included 256 scans, a receiver gain of 101, an acquisition time of 2.62 s, a relaxation delay of 4 s, a spectral width of 12,500 (20.8287 ppm), and 4 dummy scans. Automatic tuning, matching, and shimming were performed for all samples. The FIDs were multiplied by an exponential weighting function corresponding to a line broadening of 0.3 Hz prior to Fourier transformation. Automatic corrections for phase and baseline distortions were applied to the transformed spectra using TopSpin built-in processing tools.

Metabolite assignment was carried out through a multifaceted approach, which involved (i) scrutinizing existing literature data [23,24,25]; (ii) cross-referencing with the chemical shifts of metabolites cataloged in the Human Metabolome Database (HMDB), and (iii) employing the peak fitting algorithm integrated within the evaluation edition of Chenomx NMR Suite 8.4 software (Chenomx, AB, Canada).

### 2.9. NMR Data Reduction and Processing

NMR spectra underwent importation into MATLAB (R2018b; MathWorks Inc., Natick, MA, USA), where the acetate singlet at 1.90 ppm was designated as the reference signal. To reduce model complexity, the peak areas corresponding to distinct and confidently identified resonances of 29 chosen metabolites were integrated and arranged into a data matrix, featuring 12 rows (representing samples) and 29 columns (representing metabolites). Subsequently, this data matrix underwent processing utilizing the PLS toolbox version 8.9 (Eigenvector Research, Manson, WA, USA) within the MATLAB framework, enabling principal component analysis (PCA). Before analysis, the data underwent autoscaling (mean-centering followed by division of each column (variable) by the standard deviation of that column) to ensure equal influence among all metabolites within the model.

### 2.10. Acquisition of Genes Related to Ferroptosis and Iron Metabolism

Genes related to ferroptosis were obtained in the iron death pathway (map04216) in the KEGG Database (https://www.genome.jp/kegg/pathway.html, accessed on 12 December 2023) [26]. Genes related to iron metabolism and cellular iron ion homeostasis were derived from the iron uptake and transport pathways (R-HSA-917937) in the Reactome Pathway Database (https://reactome.org/, accessed on 12 December 2023) and the AmiGo2 Database (http://amigo.geneontology.org/amigo, accessed on 12 December 2023), respectively [27]. After eliminating duplicates, we obtained a total of 170 ferroptosis-related genes for use in this study (Table S1). The whole gene list was previously identified by Liu et al. [28].

### 2.11. Data Collection

RNA-Seq data (in V2 RSEM Z-score format) and clinicopathological and survival data of 382 CRC patients (TCGA Pan-Cancer Atlas) [29,30] were retrieved from the cBioPortal for Cancer Genomics interface (http://www.cbioportal.org/, accessed on 12 December 2023). RNA-Seq data were used for the correlation analysis between the expression levels of ferroptosis-related genes and uL3 (Spearman’s correlation > 0.60, *p*-value (*p*) < 0.005). Correlation coefficients were calculated before the Log2 transformation of RNA-Seq data. Log2 transformed RNA-Seq data were used for heat map, hierarchical clustering, and survival analysis [31].

### 2.12. Library Preparation and Deep Sequencing

For RNA-Seq analysis, libraries were prepared using TruSeq RNA Sample Preparation kit (Agilent Technologies, La Jolla, CA, USA), as described in [31]. High Sensitivity DNA Assay Kit on a Bioanalyzer (Agilent Technologies, La Jolla, CA, USA) and Qubit quantification platform (Qubit 2.0 Fluorometer, Life Technologies, Carlsbad, CA, USA) were used to check the quality of library templates and to normalize samples for library preparation. Libraries were sequenced on a NovaSeq6000 platform (Illumina, Inc. San Diego, CA, USA). The sequencing was carried out in collaboration with the Next Generation Facility at TIGEM (Naples, Italy). The data have been deposited in NCBIs Gene Expression Omnibus (GEO) [32]. The GEO accession number is GSE145807.

### 2.13. Computational Analysis of Deep Sequencing Data

A data analysis was performed using the pipeline already established at the Bioinformatics and Statistics Core Facility at TIGEM (Naples, Italy) [31].

Briefly, after trimming to remove adapter sequences and low-quality ends and filtering out contaminating sequences (e.g., ribosomal RNA, phIX control), reads were aligned and assigned to Human ENSEMBLE transcripts and genes (hg38 reference; RSEM version 1.2.25) [31]. The threshold for statistical significance chosen was false discovery rate (FDR) < 0.05.

### 2.14. Western Blotting (WB) 

Proteins were extracted from HCT 116^p53−/−^ and uL3ΔHCT 116^p53−/−^ cells as previously described [33]. WB analysis was performed as previously reported [9]. The membranes were challenged with Anti-SLC7A11, Anti-GPX4, Anti-Nrf2, Anti-p21, Anti-TfR1, Anti-GAPDH, Anti-HA-probe, and Anti-α-tubulin. Proteins were visualized with ECL Detection Kit according to the manufacturer’s instructions. ImageJ version 1.8.0 was used to quantify the band intensities.

### 2.15. Analysis of mRNA Stability

HCT 116^p53−/−^ cells and uL3∆HCT 116^p53−/−^ cells transfected or not with pHA-uL3 were treated with Act D (5 μg/mL) for 0, 6, 12, and 24 h. Then, total RNA was isolated, and the mRNA levels of SLC7A11 were determined by RT-qPCR using specific primers (Table 1). The relative amount of SLC7A11 mRNA without Act D treatment was set to 1, and the levels of SLC7A11 mRNA treated with Act D were calculated accordingly.

### 2.16. Cystine Uptake Assay

The cystine uptake levels were measured using the Cystine Uptake Assay Kit according to the manufacturer’s instructions. Briefly, HCT 116^p53−/−^ and uL3ΔHCT 116^p53−/−^ cells (1 × 10^4^ cells/well) were seeded into a 96-well black plate. After 24 h of plasmid transfection or erastin treatment (10 μM), cells were washed three times with cystine-free, serum-free DMEM, and then incubated with 200 μL of cystine-free, serum-free DMEM for 5 min at 37 °C. Later, cells were incubated with 200 μL of Cystine Analog Solution for 30 min at 37 °C. Then, cells were washed three times with ice-cold PBS, and then incubated with 50 μL of methanol + 200 μL of working solution for 30 min at 37 °C. Finally, fluorescence intensity was measured at λ_ex_ = 490/λ_em_ = 535 nm using a Biotek Synergy H1 Hybrid multiplate reader (Agilent Technologies, Santa Clara, CA, USA). Cystine uptake levels were expressed as the percentage of fluorescence values in treated samples with respect to that of the control (100%).

### 2.17. GSH/GSSG Determination

GSH and oxidized glutathione (GSSG) levels were measured using the GSSG/GSH Quantification Kit according to the manufacturer’s protocol. After 24 h of transfection and/or erastin treatment (10 μM), HCT 116^p53−/−^ and uL3ΔHCT 116^p53−/−^ cells (1.5 × 10^6^) were washed with ice-cold PBS, resuspended in 120 μL of HCl (10 mM), and then frozen and thawed twice for cell lysis. After the addition of 30 μL of 5% SSA, the mixture was centrifuged at 8000× *g* for 10 min at 4 °C. The supernatant was transferred to a new tube, and SSA concentration was reduced to 0.5% by the addition of ddH_2_O. For GSSG measurement, a masking solution was added to part of the samples. Then, 40 µL of samples, GSH standard solutions or GSSG standard solutions was added to the designated wells of a 96-well plate and mixed with 60 µL of Buffer solution. After 1 h incubation at 37 °C, 60 µL Substrate working solution + 60 μL of Enzyme/Coenzyme working solution were added to each well, then the plate was incubated at 37 °C for 10 min. Finally, the absorbance values of the solution in each well were measured at 405 nm using a Biotek Synergy H1 Hybrid multiplate reader. The concentration of GSH in the sample as well as GSH/GSSG ratio were calculated according to the manufacturer’s instructions.

### 2.18. MTT Assay

HCT 116^p53−/−^ and uL3ΔHCT 116^p53−/−^ cells (8 × 10^3^ cells/well) were plated in serum-containing media in 96-well plates. After drug treatment and/or cell transfection, cell viability was assessed by adding MTT as previously reported [34]. The absorbance values of the solution in each well were detected at 570 nm using a Biotek Synergy H1 Hybrid multiplate reader. All MTT experiments were performed in triplicate. Cell viability was expressed as the percentage of absorbance values in treated samples with respect to that of the control (100%).

### 2.19. Cell Cycle Analysis

HCT 116^p53−/−^ and uL3ΔHCT 116^p53−/−^ cells were seeded into 60 mm tissue culture plates at a confluency of about 50–60%. Then, cells were serum starved overnight and treated with erastin (10 μM) for 24 h. After the treatment, the cells (2 × 10^6^) were harvested and centrifuged at 400 g for 5 min, washed once with cold PBS and stained in a PI solution. Cell cycle distribution was analyzed using a BD Accuri C6 Plus flow cytometer (BD Biosciences, San Jose, CA, USA).

### 2.20. Cell Death Assay

HCT 116^p53−/−^ and uL3ΔHCT 116^p53−/−^ cells (3 × 10^5^), seeded into 60 mm tissue culture plates, were treated with erastin (10 μM) for 24 h. Then, the cells were washed with PBS, harvested by trypsinization, and washed twice with PBS. Then, cells were stained with PI and Annexin V-Alexa Fluor 488 using Tali™ Apoptosis Kit according to the manufacturer’s instructions. Briefly, cells (1 × 10^6^) were resuspended with 1× binding buffer (100 μL). Then, Annexin V-Alexa Fluor 488 (5 μL) was added, and cell were incubated for 20 min at RT in the dark. After centrifugation, cells were resuspended with 1× binding buffer (100 μL), stained with PI (1 μL), and analyzed by a BD Accuri C6 Plus flow cytometer. For each sample, at least 2 × 10^4^ events were analyzed. The percentages of Annexin V^+^/PI^−^ (early apoptosis), Annexin V^+^/PI^+^ (late apoptosis), and Annexin V^−^/PI^+^ (non-apoptotic cell death) cells were analyzed based on the manufacturer’s instructions.

### 2.21. Total Iron Measurement

Total iron was measured using the Iron Assay Kit following the manufacturer’s instructions. uL3ΔHCT 116^p53−/−^ cells (2 × 10^6^), incubated with erastin (10 μM) for 24 h, were homogenized in 5 volumes of Iron Assay Buffer. Samples were centrifuged at 16,000× *g* for 10 min at 4 °C; then, supernatants were collected and added to a 96-well plate (100 μL/well). Iron Reducer (5 μL) was added to each well, and the treated samples were incubated for 30 min at RT in the dark. Then, 100 μL of Iron Probe was added to each well, and the samples were mixed by using a horizontal shaker and incubated for 60 min at RT in the dark. Finally, the absorbance values of the solution in each well were measured at 593 nm using a Biotek Synergy H1 Hybrid multiplate reader. Total iron was expressed as the percentage of absorbance values in treated samples with respect to that of the control (100%).

### 2.22. Intracellular ROS Analysis

Intracellular ROS were evaluated by using OxiSelect™ Intracellular ROS Assay Kit following the manufacturer’s recommendations. Briefly, uL3ΔHCT 116^p53−/−^ cells were seeded onto 96-well black plates with clear bottoms for fluorometric assays (1 × 10^4^ cells/well). On the next day, cells were transfected or not for 24 h. Then, cells were treated with erastin (10 μM), and 24 h later, they were washed three times with PBS and incubated with 100 μL/well of DCFH-DA (10 μM) solution in serum-free cell culture media for 30 min at 37 °C. After removing the solution, 100 μL of PBS was added to each well, and fluorescence intensity was measured at λ_ex_ = 480/λ_em_ = 530 nm using a Biotek Synergy H1 Hybrid multiplate reader. ROS levels were expressed as the percentage of fluorescence values in treated samples with respect to that of the control (100%).

### 2.23. Statistical Analysis

Statistical analyses were performed by GraphPad Prism version 8.0 (GraphPad Software, Inc., La Jolla, CA, USA). Experiments were performed at least three times with replicate samples. Results are expressed as mean ± standard deviation (SD), unless indicated otherwise. Groups were compared with either a two-tailed Student *t*-test (for analysis of two groups) or using one-way analysis of variance (ANOVA) or two-way ANOVA followed by Dunnet’s multiple comparisons test to compare multiple groups. Significance was accepted when *p* was less than 0.05. The statistical differences of gene expression levels were analyzed by Mann–Whitney Wilcoxon test or Student *t*-test according to the distribution of the variables. The results were presented as mean ± SD of samples. We used Spearman’s and Pearson’s R correlation coefficient to assess relationships between the mRNA expression levels of uL3 and other genes. *p* < 0.05 was considered as statistically significant. For survival data, Kaplan–Meier curves were plotted and compared using a log-rank test. All tests were two-sided.

## 3. Results

### 3.1. uL3 Silencing Enhances Metastatic Potential of p53-Deleted CRC Cells in CAM Xenograft Assay

**Figure 1:**
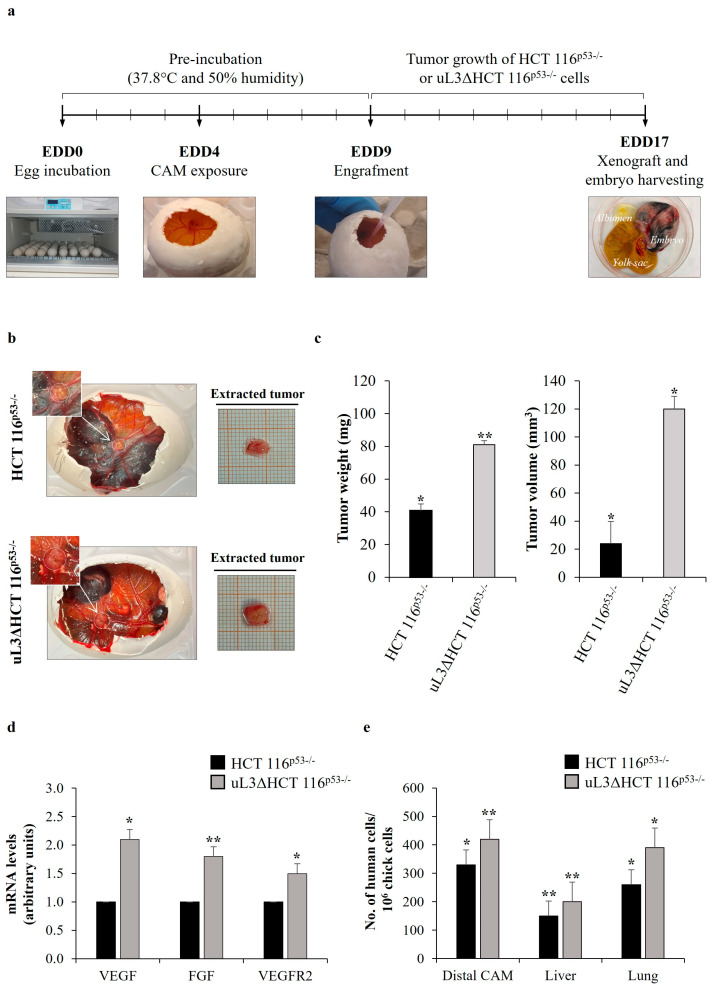
Angiogenic and metastatic potential of uL3∆HCT 116^p53−/−^ cells in Chorioallantoic Membrane (CAM) model. (**a**) General workflow of in ovo CAM assay. Fertilized eggs were incubated at 37.8 °C. Eggs were opened on egg development day (EDD) four (EDD4). After 5 days, HCT 116^p53−/−^ or uL3∆HCT 116^p53−/−^ cells in Matrigel pellets (3 × 10⁶ tumor cells per pellet) were applied to the CAM, and xenografts were allowed to develop and grow for 8 days. On EDD17, CAM xenografts were harvested, embryos were sacrificed, and chick embryo tissues and xenografts were collected for further processing. (**b**) Representative macroscopic images of HCT 116^p53−/−^ and uL3∆HCT 116^p53−/−^ cell line-derived tumors on EDD17. (**c**) Quantification of tumor weight and tumor volume of HCT 116^p53−/−^ and uL3∆HCT 116^p53−/−^ cell-derived tumors is shown. * *p* < 0.05; ** *p* < 0.01. (**d**) Expression of *VEGF*, *FGF* and *VEGFR2* in the proximal CAM of HCT 116^p53−/−^ and uL3ΔHCT 116^p53−/−^ xenografts was quantified by RT-qPCR using specific primers (Table 1). Bars represent the mean of triplicate experiments; error bars represent the standard deviation. * *p* < 0.05; ** *p* < 0.01. (**e**) qPCR quantification of human HCT 116^p53−/−^ and uL3ΔHCT 116^p53−/−^ cells in the distal CAM, liver and lung tissues using universal human Alu (h-*Alu*) primers. As internal control used to validate the presence of an equivalent amount of chick genomic DNA (gDNA), we employed chick GAPDH (ch-*GAPDH*) primers (Table 2). Bars represent the mean of triplicate experiments; error bars represent the standard deviation. * *p* < 0.05; ** *p* < 0.01.

Tumor metastases induced by drug resistance are the leading cause of cancer treatment failure [35]. We have previously demonstrated that the loss of uL3 is associated with an enhanced invasive phenotype [8]. In order to investigate the effect of uL3 status on the metastatic potential of HCT 116^p53−/−^ cells, we used the CAM of the developing chick embryo as a model. The step-by-step experimental procedure is presented in Figure 1a. Specifically, fertilized eggs were incubated for 4 days, when a hole was made in the shell. Sensitive HCT 116^p53−/−^ cells, and resistant HCT 116^p53−/−^ cells stably silenced of uL3, namely uL3ΔHCT 116^p53−/−^ cells [8], were transplanted onto the CAM on EDD9. Then, xenografts were allowed to develop and grow for 8 days on the CAM. Every day, visual inspection was used to track the formation of tumors and the vitality of chick embryos. On EDD17, CAM xenografts were harvested, weighed and measured using a digital caliper (Figure 1b,c). We observed a significant difference in the weight and volume of resected tumors between HCT 116^p53−/−^ and uL3ΔHCT 116^p53−/−^ cell line-derived tumors. Specifically, uL3ΔHCT 116^p53−/−^ xenografts showed significantly higher tumor weight and volume compared with HCT 116^p53−/−^ xenografts (Figure 1c).

A crucial event of the metastatic pathway is the recruitment of new blood vessels that provide the principal route by which cancer cells escape from the primary tumor site and spread throughout the circulation [36]. Based on this, we investigated the ability of uL3-silenced CRC cells to induce angiogenesis. Tumor angiogenesis is regulated by the production of angiogenic stimulators including members of the fibroblast growth factor (FGF) and vascular endothelial growth factor (VEGF) families [37]. For this purpose, the portion of the CAM surrounding xenografts (proximal CAM) was harvested, and total RNA was extracted. Then, the expression levels of *VEGF*, *FGF* and vascular endothelial growth factor receptor 2 (*VEGFR2*) were analyzed by RT-qPCR using specific primers (Table 1). The results showed that the expression of *VEGF*, *FGF* and *VEGFR2* in the proximal CAM of uL3ΔHCT 116^p53−/−^ xenografts was significantly increased compared to the proximal CAM of HCT 116^p53−/−^ xenografts (Figure 1d). 

CRC typically displays a specific organ colonization pattern, spreading to the liver and lungs as the most common sites of metastases [38]. Consequently, we isolated the portion of the CAM distal from the primary tumor site (distal CAM), lungs and liver from chick embryos on EDD17 to analyze metastatic tumor evolution. Metastasized human cancer cells were detected by quantitative Alu-PCR in the isolated organs of the chick embryos by using specific primers (Table 2). Figure 1e shows that uL3ΔHCT 116^p53−/−^ cells disseminated to all investigated organs including distal CAM to a greater extent than parental cells. 

These findings indicate that the silencing of uL3 increased the angiogenic activity of p53-deleted CRC cells, thus promoting metastasis formation.

### 3.2. uL3 Silencing Causes CRC Metabolic Reprogramming

Drug-resistant cancer cells acquire specific metabolic mechanisms to fight against anticancer drugs [14]. In an effort to characterize metabolic alterations associated with uL3-mediated resistance, we assessed the metabolomic profiles of sensitive HCT 116^p53−/−^ cells and resistant uL3ΔHCT 116^p53−/−^ cells. To this end, both cell lines were treated or not with 10 μM of 5-FU, and 48 h later, the samples were submitted to metabolomics studies by employing PCA for data exploration.

**Figure 2:**
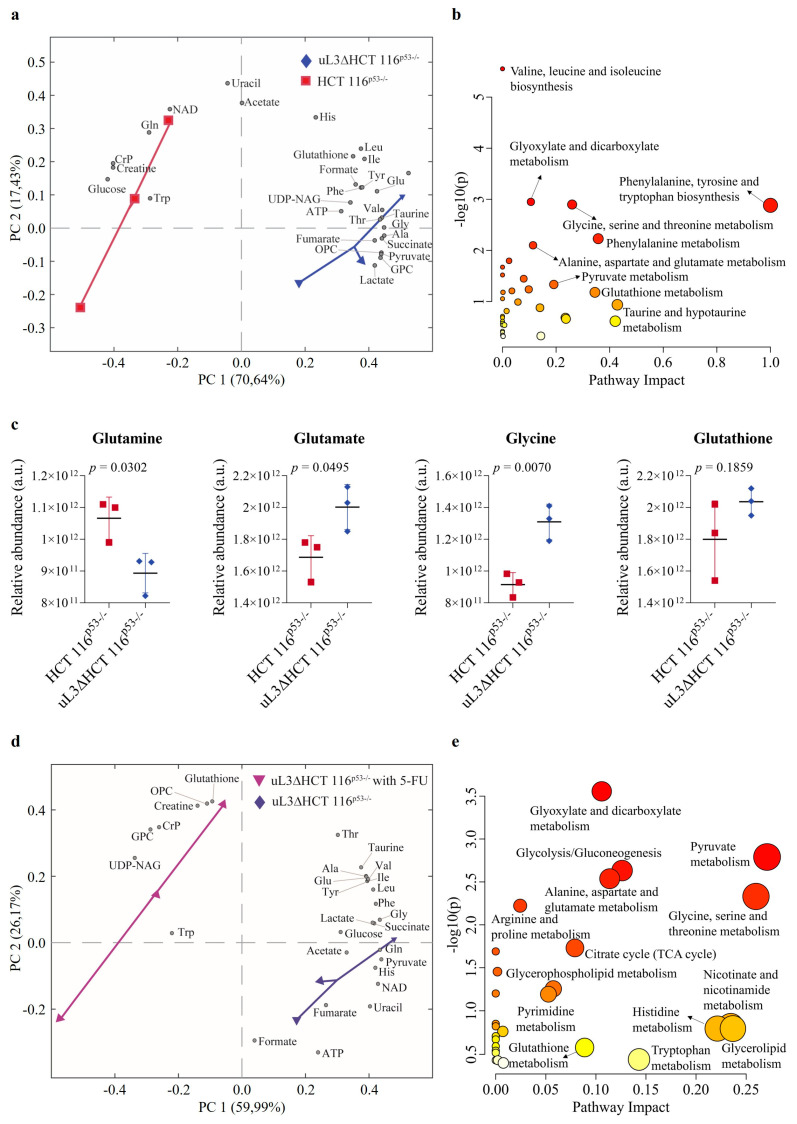
Effect of uL3 silencing on metabolite profiles. Biplot (scores plot combined with loading plot) of principal component analysis (PCA) model derived from the NMR-based metabolomic analyses of uL3ΔHCT 116^p53−/−^ cells vs. HCT 116^p53−/−^cells (**a**) or vs. 5-fluorouracil (5-FU)-treated uL3ΔHCT 116^p53−/−^ cells (**d**), along with their relative pathway analyses, respectively (**b**,**e**). Pathway analyses show all matched pathways according to p-values (*p*) (y-axis) and pathway impact values (x-axis). Circle colors represent p value from yellow (high *p*) to red (low *p*). (NAD, Nicotinamide adenine dinucleotide; CrP, Creatine Phosphate; UDP-NAG, Uridine diphosphate N-acetylglucosamine; OPC, O-phosphocholine; GPC, Glycerophosphocholine; TCA, tricarboxylic acid). (**c**) Bar graphs reporting the NMR-based relative quantification of glutamine, glutamate, glycine, and glutathione in uL3ΔHCT 116^p53−/−^ cells vs. HCT 116^p53−/−^ cells.

The PCA score plot (Figure 2a) showed clear separation between HCT 116^p53−/−^ and uL3ΔHCT 116^p53−/−^ cells. The pathway analysis revealed that AA and GSH metabolism were extremely altered in the condition of uL3 silencing (Figure 2b). 

Specifically, uL3ΔHCT 116^p53−/−^ cells showed higher amounts of glutamate and glycine, but lower levels of glutamine when compared to HCT 116^p53−/−^ cells. Of note, compared to sensitive parental cells, GSH levels were markedly elevated in uL3ΔHCT 116^p53−/−^ cells (Figure 2c). This is consistent with previous studies demonstrating that increased intracellular GSH content is critical for uL3-based chemotherapy resistance [11]. We also found that uL3ΔHCT 116^p53−/−^ cells had higher amounts of pyruvate and lactate and lower levels of glucose when compared to sensitive parental cells. Furthermore, the levels of succinate and fumarate, two Krebs cycle intermediates, were higher in resistant cells than in sensitive cells (Figure 2a).

Upon 5-FU treatment, HCT 116^p53−/−^ cells showed a strong reduction in the levels of most metabolites, indicating that 5-FU treatment induced extensive metabolic disruption (Figure S1). On the contrary, the treatment of uL3ΔHCT 116^p53−/−^ cells with 5-FU caused a significant upregulation of GSH levels, indicating that GSH metabolism is involved in uL3-mediated drug resistance (Figure 2d,e).

These data clearly indicate that uL3 silencing causes metabolic reprogramming of p53-deleted CRC cells by markedly impairing the metabolism of AAs, particularly those involved in GSH metabolism.

### 3.3. uL3 Silencing Is Associated with the Alteration of Ferroptosis-Related Gene Expression

**Figure 3:**
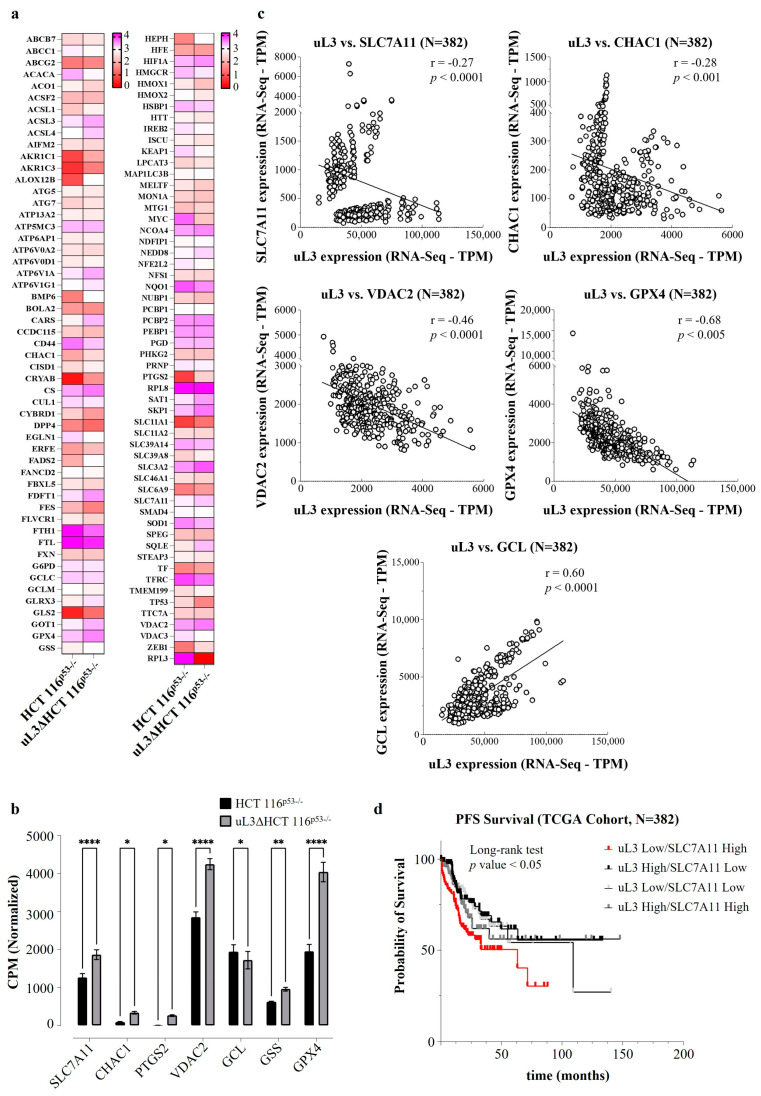
Correlation between uL3 status and the expression of ferroptosis-related genes in colorectal cancer (CRC) cells and patients. (**a**) The heatmap shows the expression of the ferroptosis-related differentially expressed genes between HCT 116^p53−/−^ and uL3ΔHCT 116^p53−/−^ cells. Pink represents significantly upregulated genes, and red represents significantly downregulated genes in the samples (FDR < 0.25; *p* < 0.05). (**b**) Expression analysis of uL3 and ferroptosis-related genes in HCT 116^p53−/−^ and uL3ΔHCT 116^p53−/−^ cells using data retrieved from RNA-Seq. Data were expressed in log-CPM (counts per million). * *p* < 0.05, ** *p* < 0.01, **** *p* < 0.0001. (**c**) Correlation between uL3 status and the expression of ferroptosis-related genes (*SLC7A11*, *CHAC1*, *VDAC2*, *GPX4*, *GCL*) in CRC patients by Spearman’s correlation (*p* < 0.005). (**d**) RNA-Seq data retrieved from TCGA database were used to stratify the patients into four groups on basis of uL3 and SLC7A11 expression (median split). PFS analysis demonstrated that patients belonging to the uL3 low/SLC7A11 high expressing group showed significantly shorter PFS (*p* < 0.05 by long-rank test).

To decipher the molecular mechanisms driven by uL3 in drug resistance, we investigated the whole transcriptomes of HCT 116^p53−/−^ and uL3∆HCT 116^p53−/−^ cells. The differential expression analysis of RNA-Seq data evidenced ferroptosis-related genes [39] as one of the most deregulated gene sets (Table S1). In particular, a total of 108 ferroptosis-related differentially expressed genes were determined, as presented in Figure 3a. Specifically, 57 were downregulated, and 51 were upregulated, as shown in Table S2. Among the most deregulated genes, we found *SLC7A11*, *CHAC1*, *PTGS2*, *VDAC2*, *GCL*, *GSS*, and *GPX4*. Specifically, the expression of *SLC7A11*, *CHAC1*, *PTGS2*, *VDAC2*, *GSS*, and *GPX4* was found to be significantly increased in resistant uL3∆HCT 116^p53−/−^ cells, while that of *GCL* decreased (Figure 3b), suggesting a crucial role of uL3 in regulating the expression of ferroptosis-related genes in p53-deleted CRC cells. 

Furthermore, utilizing a dataset of 382 CRC patients, a correlation analysis was conducted to assess whether uL3 expression correlated with ferroptosis-related genes in these patients and whether this could affect the CRC patients’ outcomes (TCGA datasets, https://www.cbioportal.org/, accessed on 12 December 2023) [30]. 

Interestingly, as previously demonstrated in the cell model (Figure 3a,b), uL3 levels showed a positive correlation with *GCL* levels and an inverse correlation with *SLC7A11*, *CHAC1*, *VDAC2*, and *GPX4* expression levels (Figure 3c). In particular, since we had previously demonstrated that uL3 is a transcriptional repressor of SLC7A11 [11], whose overexpression is associated with a chemoresistant phenotype [40], we became interested in investigating the clinical significance of the correlation between uL3 and SLC7A11 expression in the aforementioned cohort of CRC patients. We stratified the patients into four groups: uL3 high/SLC7A11 high; uL3 high/SLC7A11 low; uL3 low/SLC7A11 high; and uL3 low/SLC7A11 low expression (median split). Progression-free survival (PFS)-based analysis demonstrated that patients belonging to the uL3 low/SLC7A11 high expressing group showed significantly shorter PFS, extending no more than 63 months for 90% of this subgroup (Figure 3d). Conversely, PFS was significantly better in the other uL3 groups (median 63 months vs. 109 months; *p* = 0.0027 by long-rank test; Figure 3d), demonstrating unequivocally that a poor outcome is linked to uL3 downregulation concurrent with high expression of SLC7A11.

Taken together, these findings, obtained by analyzing the transcriptomes of a large cohort of CRC patients, suggest that uL3 may be implicated in cancer resistance through the regulation of ferroptosis-related genes. These findings imply that ferroptotic agent susceptibility may be used to treat resistant uL3∆HCT 116^p53−/−^ cells.

### 3.4. uL3 Is a Negative Regulator of SLC7A11 mRNA Stability

**Figure 4:**
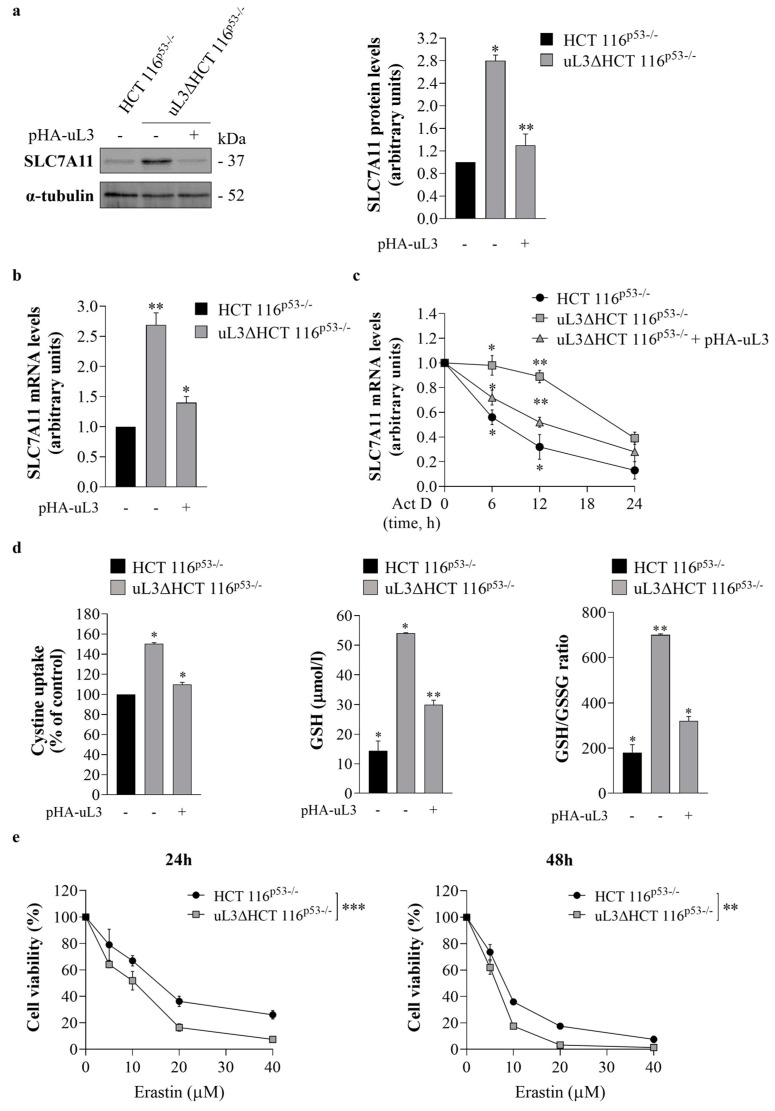
Effect of uL3 silencing on SLC7A11 expression and erastin cytotoxic activity. (**a**) Protein extracts from HCT 116^p53−/−^ cells and uL3∆HCT 116^p53−/−^ cells transfected or not with pHA-uL3 were analyzed by western blotting (WB) with anti-SLC7A11 antibody; α-tubulin was used as loading control. Full-length blots are shown in Figure S7. The graph shows the relative densitometric analyses, expressed as arbitrary units. Bars represent the mean of triplicate experiments; error bars represent the standard deviation. * *p* < 0.05, ** *p* < 0.01 vs. HCT 116^p53−/−^ cells set at 1. (**b**) RT-qPCR of total RNA extracted from HCT 116^p53−/−^ cells and uL3∆HCT 116^p53−/−^ cells transfected or not with pHA-uL3 using specific primers for *SLC7A11* (Table 1). * *p* < 0.05, ** *p* < 0.01 vs. HCT 116^p53−/−^ cells set at 1. (**c**) HCT 116^p53−/−^ cells and uL3∆HCT 116^p53−/−^ cells transfected or not with pHA-uL3 were treated with actinomycin D (Act D) (5 μg/mL). At the indicated time points (0, 6, 12 and 24 h), total RNA was isolated, and mRNA levels of *SLC7A11* and *β-actin* were determined by RT-qPCR by using specific primers (Table 1). The relative amount of *SLC7A11* mRNA in untreated cells was set to 1, and the levels of *SLC7A11* mRNA in cells treated with Act D were calculated accordingly. * *p* < 0.05, ** *p* < 0.01 vs. untreated cells. (**d**) Evaluation of cystine uptake, reduced glutathione (GSH) levels and GSH/ oxidized glutathione (GSSG) ratio in HCT 116^p53−/−^ cells and uL3∆HCT 116^p53−/−^ cells transfected or not with pHA-uL3 for 24 h. * *p* < 0.05, ** *p* < 0.01 vs. HCT 116^p53−/−^ cells. (**e**) HCT 116^p53−/−^ and uL3∆HCT 116^p53−/−^ cells were treated or not with different concentration of erastin (from 5 to 40 µM) for 24 and 48 h. After incubation, cell viability was evaluated using the MTT assay. Cell viability of untreated cells was set to 100%. Bars represent the mean of triplicate experiments; error bars represent the standard deviation. ** *p* < 0.01, *** *p* < 0.001 uL3ΔHCT 116^p53−/−^ cells vs. HCT 116^p53−/−^ cells.

To validate transcriptome data, the intracellular amount of SLC7A11 was analyzed at the protein and mRNA levels upon alteration of uL3 expression levels. For this purpose, total RNA and protein extracted from sensitive HCT 116^p53−/−^ cells and resistant uL3ΔHCT 116^p53−/−^ cells, untransfected or transiently transfected with a plasmid encoding recombinant uL3 (pHA-uL3) for 24 h (Figures S2 and S6), were analyzed by RT-qPCR with specific primers (Table 1) and WB, respectively. The results demonstrated that uL3ΔHCT 116^p53−/−^ cells showed a higher amount of SLC7A11 protein and mRNA compared to the parental cell line; the restoration of uL3 was associated with a reduction in SLC7A11 expression levels (Figure 4a,b). These data indicate that SLC7A11 expression is regulated by intracellular levels of uL3.

To guarantee appropriate activity of SLC7A11 in maintaining redox homeostasis, the expression of SLC7A11 is subjected to multiple regulatory mechanisms, including those at the post-transcriptional level, to control its mRNA level [17]. In order to investigate the potential role of uL3 in the control of *SLC7A11* mRNA stability, sensitive HCT 116^p53−/−^ cells and resistant uL3ΔHCT 116^p53−/−^ cells, untransfected or transiently transfected with pHA-uL3 for 24 h, were treated with Act D (5 μg/mL) to inhibit nascent mRNA synthesis. Total RNA was obtained from cells at the indicated times (0, 6, 12 and 24 h), and *SLC7A11* mRNA levels were analyzed by RT-qPCR using specific primers (Table 1). The results showed that in sensitive HCT 116^p53−/−^ cells, the amount of *SLC7A11* transcript was lower than that in resistant uL3∆HCT 116^p53−/−^ cells at all tested time points (Figure 4c). In uL3ΔHCT 116^p53−/−^ cells, the levels of *SLC7A11* mRNA were approximately 50% increased compared to those in HCT 116^p53−/−^ cells after 6 h of Act D treatment. The restoration of uL3 was associated with a reduction in *SLC7A11* transcript half-life (Figure 4c). These results indicate that uL3 acts as a negative regulator of *SLC7A11* mRNA stability. In fact, in the absence of uL3, the observed upregulation of *SLC7A11* mRNA levels could be partially due to an increase in *SLC7A11* mRNA stability.

To verify that uL3 affects SLC7A11 activity by controlling its expression, uL3∆HCT 116^p53−/−^ cells were transiently transfected with pHA-uL3. Twenty-four hours later, cystine uptake and GSH content were detected. The results showed that, upon uL3 restoration, cystine uptake was strongly inhibited (Figure 4d). Accordingly, a reduction in GSH content as well as the GSH/GSSG ratio compared to untreated cells was observed (Figure 4d).

### 3.5. uL3 Silencing Promotes CRC Cells to Ferroptosis

It has been demonstrated that higher SLC7A11 expression in some types of tumors creates a targetable vulnerability, as these tumors are highly dependent on this transporter and should be more sensitive to oxidative stress [41]. 

Several compounds have been identified as pharmacological SLC7A11 inhibitors. Among them is erastin, a small molecule that can inhibit SLC7A11 activity, leading to GSH biosynthesis impairment and redox imbalance [21,42]. With the aim of examining the effect of erastin on cell viability, sensitive HCT 116^p53−/−^ cells and resistant uL3∆HCT 116^p53−/−^ cells were treated with different concentrations (from 5 to 40 μM) of erastin. Then, cytotoxicity was assessed by the MTT assay after 24 and 48 h of treatment. According to earlier research [43], HCT 116^p53−/−^ cells showed sensitivity to erastin, and cell viability was decreased in a dose- and time-dependent manner (Figure 4e). Of note, erastin exhibited cytotoxic activity also in resistant uL3∆HCT 116^p53−/−^ cells (Figure 4e).

**Figure 5:**
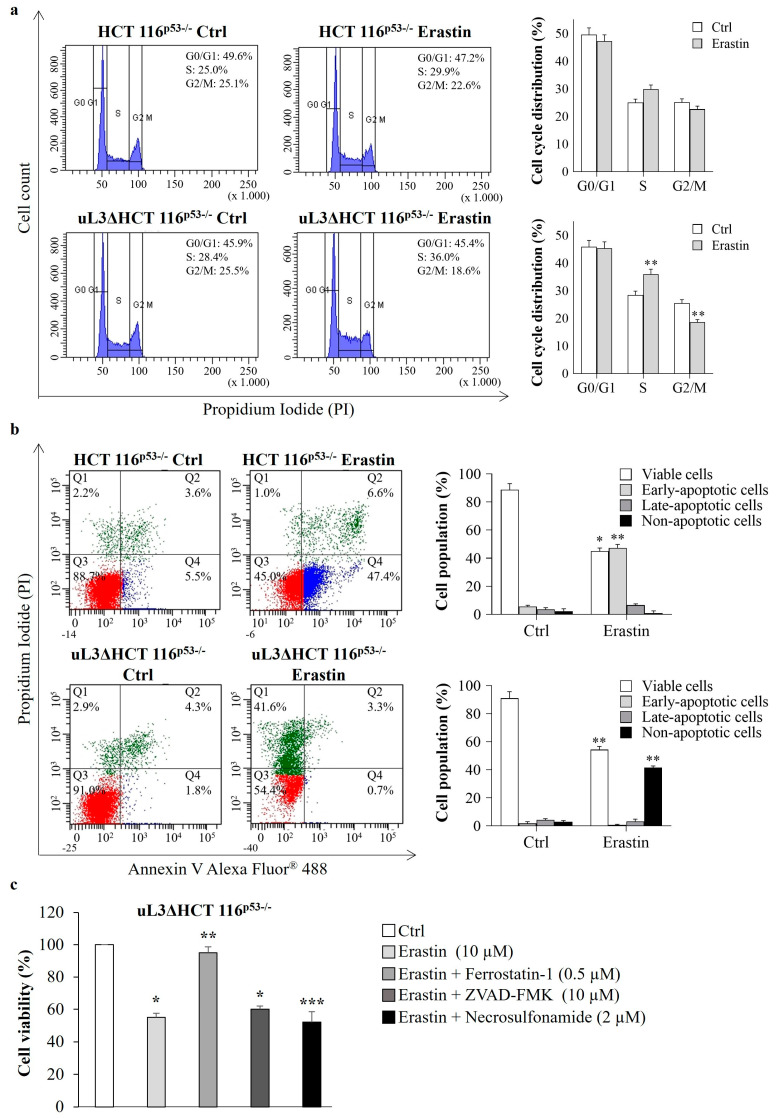
Effect of erastin treatment on cell cycle distribution and cell death. (**a**) Representative FACS histograms of propidium iodide (PI)-stained HCT 116^p53−/−^ and uL3∆HCT 116^p53−/−^ cells treated or not with erastin (10 µM) for 24 h. The bar diagram shows the percentage of cells in each phase of the cell cycle. ** *p* < 0.01 vs. untreated cells. (**b**) Representative dot blots of Annexin V-Alexa Fluor 488 and PI-stained HCT 116^p53−/−^ and uL3ΔHCT 116^p53−/−^ cells treated or not with erastin (10 µM) for 24 h. The bar diagram shows the percentage of viable cells (Annexin V^−^/PI^−^), early apoptotic cells (Annexin V^+^/PI^−^), late-apoptotic cells (Annexin V^+^/PI^+^) and non-apoptotic cells (Annexin V^−^/PI^+^). * *p* < 0.05, ** *p* < 0.01 vs. untreated cells. (**c**) uL3∆HCT 116^p53−/−^ cells were treated with erastin (10 µM) in the absence or presence of ferrostatin-1 (a ferroptosis inhibitor), Z-VAD-FMK (an apoptosis inhibitor), and necrosulfonamide (a necroptosis inhibitor) for 24 h. Then, cell viability was evaluated using the MTT assay. Cell viability of untreated cells was set to 100%. Bars represent the mean of triplicate experiments; error bars represent the standard deviation. * *p* < 0.05, ** *p* < 0.01, *** *p* < 0.001 vs. untreated cells.

To investigate the underlying mechanism that contributes to erastin-mediated cytotoxicity, alterations in the cell cycle distribution were analyzed. To this end, HCT 116^p53−/−^ and uL3∆HCT 116^p53−/−^ cells were treated with 10 µM of erastin for 24 h. Then, cell cycle distribution was monitored by flow cytometry. As shown in Figure 5a, erastin treatment of HCT 116^p53−/−^ cells did not alter the cell cycle. The exposure of uL3-silenced CRC cells to erastin resulted only in a little accumulation of cells in the S phase of the cell cycle (from about 28.4% to about 36.0%). Accordingly, the number of cells in G2/M phase decreased from 25.5% to 18.6% of the total population (Figure 5a).

Next, we determined whether erastin decreased cell survival through the induction of apoptosis. To this end, Annexin V analysis was performed. HCT 116^p53−/−^ and uL3∆HCT 116^p53−/−^ cells were treated with 10 µM of erastin and 24 h later were assessed with Annexin V-Alexa Fluor 488. We observed that the treatment of HCT 116^p53−/−^ cells with erastin significantly increased the percentage of early apoptotic cells (Annexin V^+^ and PI^−^) from 5.5% in untreated cells to 47.4% in treated cells (Figure 5b). Of note, erastin failed to induce apoptosis in uL3∆HCT 116^p53−/−^ cells (Figure 5b), as in these cells we observed an increase in non-apoptotic cell death (from 2.9% to 41.6%).

In order to shed a light on the death signaling pathways activated in uL3-silenced CRC cells upon erastin treatment, we exposed CRC cells to erastin in the absence or presence of different inhibitors, including the ferroptosis inhibitor ferrostatin-1 [21], the apoptosis inhibitor Z-VAD-FMK and the necroptosis inhibitor necrosulfonamide. Figure 5c shows that ferrostatin-1 inhibited erastin-induced cell death, while in the presence of Z-VAD-FMK or necrosulfonamide, erastin was able to act; we found a comparable percentage of dead cells also in the presence of these inhibitors. Thus, silencing of uL3 from HCT 116^p53−/−^ cells promotes erastin-induced cell death that occurs through ferroptosis. 

**Figure 6:**
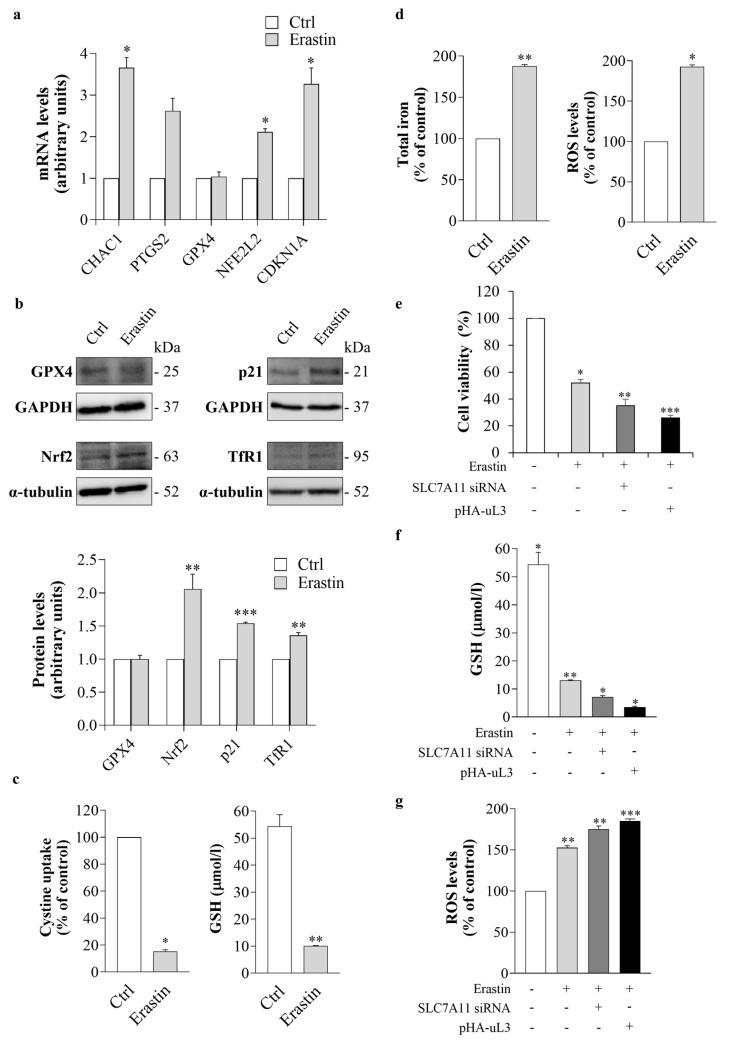
Effect of uL3 silencing on erastin-induced ferroptosis. (**a**) uL3ΔHCT 116^p53−/−^ cells were treated or not with erastin (10 µM) for 24 h. Then, total RNA was subjected to RT-qPCR with specific primers for the indicated genes (Table 1). Bars represent the mean of triplicate experiments; error bars represent the standard deviation. * *p* < 0.05 vs. control cells set at 1. (**b**) uL3∆HCT 116^p53−/−^ cells were treated or not with erastin (10 µM) for 24 h. Then, protein extracts were analyzed by WB with the indicated antibodies. GAPDH and α-tubulin were used as loading control. Full-length blots are shown in Figure S8. The graph shows the relative densitometric analyses, expressed as arbitrary units. Bars represent the mean of triplicate experiments; error bars represent the standard deviation. ** *p* < 0.01, *** *p* < 0.001 vs. control cells set at 1. (**c**,**d**) Evaluation of cystine uptake, GSH levels, total iron content and reactive oxygen species (ROS) production in uL3ΔHCT 116^p53−/−^ cells after treatment with erastin (10 µM) for 24 h. * *p* < 0.05, ** *p* < 0.01 vs. control cells. (**e**) uL3∆HCT 116^p53−/−^ cells were transfected or not with SLC7A11 siRNA or pHA-uL3 and treated with erastin (10 µM) for 24 h. Then, cell viability was evaluated using the MTT assay. Cell viability of untreated cells was set to 100%. Bars represent the mean of triplicate experiments; error bars represent the standard deviation. * *p* < 0.05, ** *p* < 0.01, *** *p* < 0.001 vs. control cells. (**f**,**g**) Evaluation of GSH levels and ROS production in uL3ΔHCT 116^p53−/−^ cells transfected or not with SLC7A11 siRNA or pHA-uL3 and treated with erastin (10 µM) for 24 h. * *p* < 0.05, ** *p* < 0.01, *** *p* < 0.001 vs. control cells.

To confirm at the molecular level that uL3 silencing sensitizes HCT 116^p53−/−^ cells to ferroptosis, we investigated whether erastin treatment of uL3∆HCT 116^p53−/−^ cells could induce changes in the transcript levels of some key ferroptosis-related genes. To this end, uL3∆HCT 116^p53−/−^ cells were treated with 10 µM of erastin. Twenty-four hours later, total RNA was extracted from untreated and treated cells and analyzed by RT-qPCR using specific primers for the indicated genes (Table 1). Genetic biomarkers associated with cells undergoing ferroptosis include increases in *CHAC1* and *PTGS2* mRNA expression [44]. As shown in Figure 6a, erastin treatment caused a strong increase in the expression levels of both *CHAC1* and *PTGS2* transcripts. The expression of glutathione peroxidase 4 (GPX4), a key antioxidant enzyme whose inactivation may increase susceptibility to ferroptosis expression [44], was not significantly altered upon erastin treatment, whereas an upregulation of *NFE2L2* transcript, which encodes for Nuclear factor erythroid 2-like 2 (Nrf2), a key transcription factor that regulates the expression of detoxification and antioxidant genes during oxidative stress [44], was observed. Of interest, erastin treatment strongly affected the amount of *CDKN1A* mRNA, encoding for cyclin kinase inhibitor p21, a well-known negative regulator of cell cycle progression whose expression can be enhanced upon erastin treatment (Figure 6a) [45]. 

Next, we assessed the ferroptotic process at the protein level by WB. In line with the results of RT-qPCR, we did not observe alterations in the expression levels of GPX4 protein upon erastin treatment, while Nrf2 and p21 levels were significantly higher compared to untreated cells (Figure 6b). We also analyzed the expression of Transferrin receptor protein 1 (TfR1), a transmembrane glycoprotein that promotes iron import from transferrin into cells. Several studies have reported that TfR1 upregulation is associated with the induction of ferroptosis [46]. According to this, Figure 6b shows that, in uL3ΔHCT 116^p53−/−^ cells treated with erastin, the expression levels of TfR1 were enhanced compared to control cells.

Next, we monitored the cystine uptake and the intracellular levels of GSH, which is essential for preventing ROS accumulation and suppressing ferroptosis [47]. Our results demonstrated that in erastin-treated cells, cystine uptake was strongly inhibited (up to 80%), and GSH content as well as the GSH/GSSG ratio were significantly lower than in untreated cells (Figure 6c and Figure S3). Consistent with these data, in erastin-treated uL3ΔHCT 116^p53−/−^ cells, the intracellular iron and ROS content were increased compared to untreated cells (Figure 6d).

### 3.6. uL3 Potentiates the Cytotoxic Effect of Erastin

It has been reported that the inhibition of SLC7A11 could enhance the cytotoxic activity of erastin [48,49].

The identification of uL3 as a negative regulator of SLC7A11 expression prompted us to explore the possibility of rescuing uL3 to potentiate the inhibitory effects of erastin on SLC7A11 activity. To this end, uL3ΔHCT 116^p53−/−^ cells were transiently transfected with pHA-uL3 (Figures S2 and S6) or siRNA against *SLC7A11* (Figure S4) as control. Twenty-four hours later, untransfected and transfected cells were treated with 10 µM of erastin for 24 h, and then cytotoxicity was assessed by MTT assay. As attended, the results shown in Figure 6e demonstrated that erastin treatment upon *SLC7A11* silencing was associated with increased cell death compared with treatment of cells with erastin alone. In line with our data, uL3, as a negative regulator of *SLC7A11*, enhanced erastin cytotoxicity. In fact, upon the combined treatment, the cell viability was strongly reduced compared with cells exposed to erastin alone (Figure 6e). Consistent with these findings, the rescue of uL3 decreased the erastin-mediated GSH depletion along with GSH/GSSG ratio reduction and increased erastin-mediated ROS induction (Figure 6f,g and Figure S3). These data demonstrated the potential enhancement of erastin-based cancer treatment by uL3.

### 3.7. Erastin Plus uL3 Inhibits Tumor Growth and Metastasis Formation in CAM Xenograft Assay

**Figure 7:**
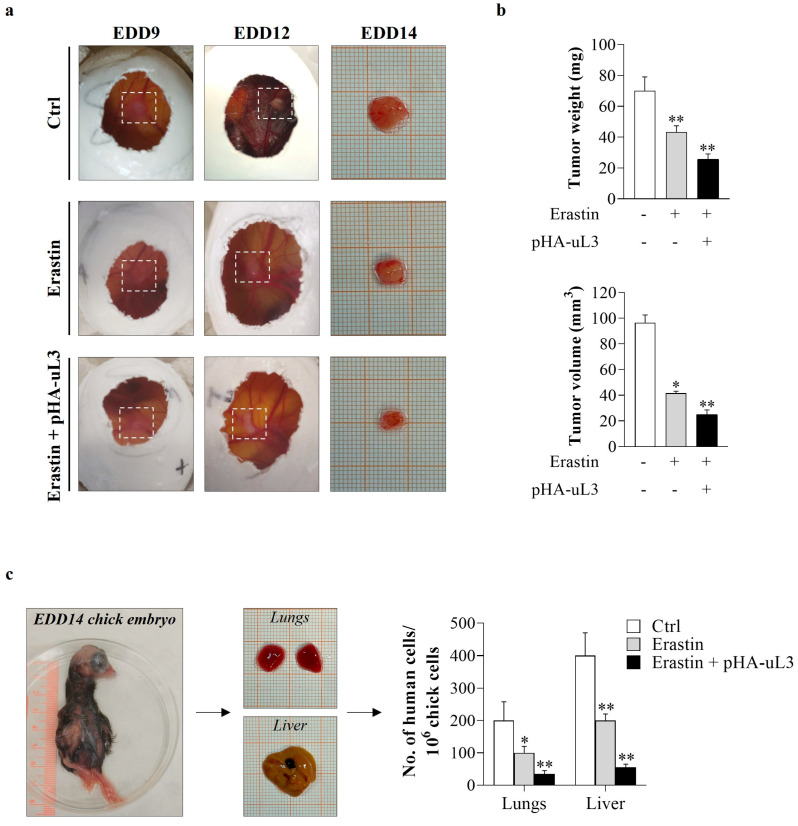
Antitumor effects of erastin plus uL3 in CAM model. uL3∆HCT 116^p53−/−^ cells, untransfected or transfected with pHA-uL3, were engrafted onto CAM surface on EDD9 and then treated or not with erastin (10 µM). (**a**) Macroscopic images of uL3∆HCT 116^p53−/−^ cell line-derived tumors were captured on EDD9, 12 and 14. Selected areas of CAM in which uL3∆HCT 116^p53−/−^ cells were engrafted are shown. (**b**) On EDD14, excised tumors were weighed and measured using a digital caliper in order to determine the effect of the treatments on tumor growth. * *p* < 0.05, ** *p* < 0.01 vs. untreated cells-derived tumors. (**c**) Representative images of EDD14 chick embryo and dissected lungs and liver. gDNA was extracted and analyzed by qPCR using specific primers for h-*Alu*. As internal control used to validate the presence of equivalent amount of chick gDNA, we employed ch-*GAPDH* primers (Table 2). * *p* < 0.05, ** *p* < 0.01 vs. control cells.

In order to evaluate the effect of the combined treatment erastin plus uL3 on tumor growth and metastasis in cell line-derived xenografts, we performed the CAM assay. uL3ΔHCT 116^p53−/−^ cells were transiently transfected with pHA-uL3 (Figure S5). Twelve hours later, untransfected and transfected cells were engrafted onto the CAM surface on EDD9 and exposed to erastin (10 µM). Five days after engraftment, the tumors were removed, weighed, and measured using a digital caliper. A significant difference in the growth of resected uL3ΔHCT 116^p53−/−^ cell line-derived tumors was observed (Figure 7a). Specifically, erastin-treated uL3ΔHCT 116^p53−/−^ xenografts showed significantly lower tumor weight and volume compared with untreated cells (Figure 7b). Of note, uL3ΔHCT 116^p53−/−^ xenografts derived from cells exposed to the combined treatment erastin plus uL3 showed a further reduction in tumor weight and volume compared to xenografts derived from untreated and erastin-treated cells (Figure 7b).

Thus, to investigate the effect of the combined treatment erastin plus uL3 on metastasis, we analyzed the metastatic potential of uL3-silenced CRC cells by quantitative Alu-PCR in the isolated lungs and livers of the chick embryos by using specific primers (Table 2). Results obtained showed that metastasis formation in the chick livers and lungs was strongly hampered by erastin plus uL3 treatment compared to erastin alone (Figure 7c).

Taken together, these findings highlight the potential application of a novel treatment approach by using erastin combined with uL3 as an antiproliferative and antimetastatic strategy for resistant CRC cells devoid of p53 and expressing low levels of uL3 and high levels of SLC7A11.

## 4. Discussion

Understanding the mechanisms involved in the progression of drug resistance and metastatic formation may guide the development of effective therapeutic strategies to reduce CRC-related death. 

We have previously produced a p53-deleted CRC cell line silenced of uL3, resulting in resistance to the most common anticancer drugs, and extensively detailed the molecular mechanisms underlying chemoresistance. Moreover, our previous data indicated that uL3 silencing remarkably promotes EMT in CRC cells, thus enhancing their migration and invasion ability [8,9,10]. 

Several studies demonstrated that the abnormal expression of specific ribosomal proteins was also associated with high invasive and metastatic potential in various cancers. To date, eS7 (RPS7) silencing increased the migration and invasion capacity of ovarian cancer cells [50]; the downregulation of uL15 (RPL27A) reduced migration and invasion in breast cancer cells [51]; and a higher amount of eL15 (RPL15) promoted metastasis in circulating tumor cells from breast cancer patients [52]. Wei et al. have reported a positive correlation of eL34 (RPL34) expression with tumor stage and metastasis in pancreatic cancer [53].

Here, to more deeply evaluate the cancer malignancy and metastatic potential of CRC cells silenced of uL3, we employed the CAM model, an in vivo system widely used to study angiogenesis and tumor invasion [54]. Our results demonstrated that tumors lacking uL3 showed a more aggressive cancer phenotype displaying more vascularization and a higher propensity to spread to the liver and lungs (Figure 1). Our results are in line with recent findings showing that the overexpression of uL3 mediated by dual oxidase 2 (DUOX2) silencing strongly reduced the metastatic capability of CRC cells [55].

In recent years, growing efforts have been directed toward the study of the metabolic reprogramming of cancer cells as a hallmark of tumor development, including invasion, metastasis, and chemoresistance [14]. Here, we investigated the metabolic changes and analyzed the transcriptome of CRC cells silenced of uL3 to elucidate biochemical and genetic alterations with the goal of identifying new druggable targets. The most altered metabolites in uL3-silenced CRC cells were mainly involved in glutamate, glycine and GSH metabolism. Moreover, glucose levels were much lower in uL3-silenced CRC cells compared to parental cells, whereas pyruvate and lactate levels were significantly higher, indicating Warburg-like metabolic alterations (Figure 2a–c) [56]. Consistent with our data, recent studies have highlighted that the abnormal expression of other ribosomal proteins (e.g., uS3 and eS7) is strictly associated with the dysregulation of specific metabolic enzymes related to the Warburg phenotype in CRC cells [57,58]. Moreover, uL3-silenced CRC cells were characterized by reduced levels of glutamine compared to parental cells (Figure 2a–c). High glutamine consumption is a characteristic of many cancers, and this finding has also been confirmed clinically; in fact, the content of plasma glutamine in individuals with cancer is considerably lower than in healthy ones. In cancer cells, glutamine is converted into tricarboxylic acid cycle metabolites to maintain mitochondrial function in condition of aerobic glycolysis [59]. According to the metabolic hallmarks of malignant progression and aggressiveness in several cancers [60], we also found abnormal choline metabolism and high levels of succinate and fumarate in resistant cells compared to sensitive ones (Figure 2a,b).

When CRC cells silenced of uL3 were treated with 5-FU, a greater increase in GSH expression was observed, suggesting that “redox resetting” enables these cells to develop drug resistance (Figure 2d,e). GSH biosynthesis requires cysteine as a rate-limiting AA since it is found in much lower abundance within the cell compared to glutamate and glycine. Cysteine availability is regulated by the activity of SLC7A11, a subunit of cystine/glutamate antiporter system xc^−^ [17]. As cancer cells exhibit higher intracellular ROS content than normal cells, SLC7A11-mediated uptake of cystine is critical for the maintenance of cancer cell redox balance through the production of GSH. According to this, SLC7A11 is highly expressed in most cancers, including CRC, and its overexpression is closely associated with oxidative stress block, chemoresistance and poor outcome of cancer patients [40]. SLC7A11 is also known as a key regulator of ferroptosis, a type of programmed cell death dependent on iron and characterized by the accumulation of lipid peroxides [18]. Significant antitumor effects obtained by targeting the SLC7A11-GSH axis have been observed in various types of human cancers [61], and accumulated studies have shown that the induction of ferroptosis could reverse drug resistance [18].

We found that, in our cellular system, SLC7A11 expression levels depend on uL3 intracellular amounts (Figure 4a,b). In particular, we demonstrated that uL3 acts as a negative regulator of *SLC7A11* mRNA stability (Figure 4c). Accordingly, SLC7A11 expression was upregulated in uL3-silenced CRC cells at both the mRNA and protein levels, resulting in higher cystine uptake and GSH content than in the parental cell line (Figure 4d). Of note, the ectopic expression of uL3 prevented SLC7A11 upregulation as well as the increase in cystine uptake and GSH content (Figure 4a–d). Starting from these data and in view of the results from transcriptomic analysis, showing a significant dysregulation of 108 ferroptosis-related genes in the absence of uL3 (Figure 3a,b), we hypothesized that the chemoresistance observed in these cells might depend on high SLC7A11 expression, which in turn is associated with high levels of GSH, and that these cells could be sensitive to the induction of ferroptosis.

Targeting SLC7A11 to induce ferroptosis could have important translational value in CRC treatment since our results demonstrated that low expression of uL3 and high levels of SLC7A11 were associated with a poor outcome in patients with CRC (Figure 3d). Among the compounds interfering with system xc^−^ and causing ferroptosis, erastin is one of the first identified [42]. It directly inhibits the activity of system xc^−^, causing a reduction in GSH production that ultimately leads to accelerated ROS production and ferroptosis [21]. Our results showed that erastin inhibited cell proliferation and induced apoptosis in CRC cells expressing uL3, as previously reported [62]. Interestingly, in the absence of uL3, erastin affected cell proliferation but failed to trigger apoptosis (Figure 4e and Figure 5b,c). At the molecular level, these effects were associated with a decrease in the activity of the cystine/glutamate antiporter SLC7A11 along with a reduction in GSH content (Figure 6c), an increase in ROS levels (Figure 6d), and the upregulation of specific ferroptotic markers, including CHAC1 (Figure 6a). It has been shown that CHAC1 overexpression can induce GSH depletion and enhance ferroptosis [63]. Moreover, upon erastin treatment, TfR1 expression levels were augmented (Figure 6b), which in turn increased intracellular iron levels (Figure 6d). All these data clearly demonstrate that erastin induces ferroptosis in uL3-silenced CRC cells and reveal that, depending on the uL3 status, erastin may selectively activate different cell death pathways. 

Numerous studies have been conducted on the function of SLC7A11 in cancer cell migration, proliferation, and chemoresistance [64,65]. SLC7A11 inhibition resulted in selective killing of cancer cells in vitro and tumor growth inhibition in vivo [66]. Moreover, tumor metastasis was markedly reduced in vivo by sulfasalazine, an inhibitor of SLC7A11 function [67].

Starting from these notions and our data indicating a negative correlation between uL3 expression and SLC7A11 expression (Figure 3 and Figure 4a–c), we investigated the antiproliferative activity of a combined treatment of erastin plus a plasmid encoding uL3 in uL3-silenced CRC cells. Notably, the combination treatment caused a strong reduction in cell viability compared to erastin alone, associated with a marked decrease in GSH content and an increase in ROS levels (Figure 6e–g). The antiproliferative activity and antimetastatic effects of the combined treatment were also investigated in uL3-silenced CRC cell line-derived tumors in the CAM model. The results showed a substantial decrease in the weight and volume of CAM tumors derived from uL3-silenced CRC cells treated with erastin in combination with uL3 compared to those treated with erastin alone (Figure 7a,b). Metastatic tumor evolution was also analyzed by using quantitative Alu-PCR for the detection and quantification of disseminated human CRC cells in the isolated organs of the chick embryos. We found that the combination therapy resulted in a significant decrease in human cell dissemination compared to treatment with erastin alone (Figure 7c). These data indicate that erastin plus uL3-mediated SLC7A11 inhibition reduced the metastatic capacity of resistant uL3-silenced CRC cells. 

## 5. Conclusions

Overall, our study provides novel insights into the molecular pathogenesis of CRC and proposes a novel combined therapy based on erastin plus uL3. This strategy could be used to establish individualized therapy by examining uL3, SLC7A11 and p53 profiles in tumors to yield better clinical outcomes.

## Figures and Tables

**Table 1:** Oligonucleotide sequences used in Quantitative Reverse Transcription Polymerase Chain Reaction (RT-qPCR) analysis.

Transcript	Accession Number	Primer	Sequence 5′-3′	Amplicon Size
*CHAC1*	NM_024111.6	Fw	ACCTTGAATACTTGCTGCGTCTGG	187 bp
		Rv	CCTGATGTCCACATGAGCACTCC	
*PTGS2*	NM_000963.4	Fw	TGGTCTGGTGCCTGGTCTGATG	124 bp
		Rv	CCTGCTTGTCTGGAACAACTGCTC	
*GPX4*	NM_002085.5	Fw	CCGCTGTGGAAGTGGATGAAGATC	102 bp
		Rv	GCAGCCGTTCTTGTCGATGAGG	
*SLC7A11*	NM_014331.4	Fw	GGCTCCATGAACGGTGGTGTG	119 bp
		Rv	GCTGGTAGAGGAGTGTGCTTGC	
*NFE2L2*	NM_006164.5	Fw	ATGCTTTGTACTTTGATGACTGC	101 bp
		Rv	CGTTTCAGTCACTTGTTCCT	
*CDKN1A*	NM_001291549.3	Fw	CCTCAAATCGTCCAGCGACCTT	393 bp
		Rv	CATTGTGGGAGGAGCTGTGAAA	
*ACTB*	NM_001101.5	Fw	TCCCTGGAGAAGAGCTACG	131 bp
		Rv	GTAGTTTCGTGGATGCCACA	
ch-*VEGF*	NM_205042.3	Fw	GGAGTTGTCGAAGGCTGCT	63 bp
		Rv	TTGATAACTTCGTTGGGCTTC	
ch-*FGF*	NM_205433.2	Fw	TTCTTCCTGCGCATCAAC	325 bp
		Rv	GGATAGCTTTCTGTCCAG	
ch-*VEGFR2*	NM_001004368.2	Fw	GGGGAAGATGTACTCGGTGA	60 bp
		Rv	CATCCATGTTCAAACATCACAA	

ch = Chick.

**Table 2:** Oligonucleotide sequences used in quantitative Alu-PCR analysis.

Gene	Primer	Sequence 5′-3′
h-*Alu*	Fw	ACGCCTGTAATCCCAGGACTT
	Rv	TCGCCCAGGCTGGCTGGGTGCA
ch-*GAPDH*	Fw	GAGGAAAGGTCGCCTGGTGGATCG
	Rv	GGTGAGGACAAGCAGTGAGGAACG

## Data Availability

All data generated during this study are included either in this article or in the Supplementary Materials.

## Supplementary Materials

The following supporting information can be downloaded at: https://www.mdpi.com/article/10.3390/antiox13070757/s1, Figure S1: Effect of 5-FU treatment on metabolite profiles of HCT 116^p53−/−^ cells; Figure S2: HA-uL3 expression levels in uL3∆HCT 116^p53−/−^ cells transfected with pHA-uL3 by WB; Figure S3: Evaluation of GSH/GSSG ratio; Figure S4: SLC7A11 expression levels in uL3∆HCT 116^p53−/−^ cells transfected with SLC7A11 siRNA; Figure S5: HA-uL3 expression levels in uL3∆HCT 116^p53−/−^ cells transfected with pHA-uL3 by RT-qPCR; Figure S6: Full-length blots of Figure S2; Figure S7: Full-length blots of Figure 4a and Figure S4; Figure S8: Full-length blots of Figure 6b; Table S1: Ferroptosis-related gene set; Table S2: Differentially expressed ferroptosis-related genes.
