# Supplementary material for: uL3 Regulates Redox Metabolism and Ferroptosis Sensitivity of p53-Deleted Colorectal Cancer Cells
Source: Antioxidants (Basel). 2024 Jun 22;13(7):757. doi: 10.3390/antiox13070757 (PMC11274089; doi:10.3390/antiox13070757)
Supplement: Supplementary file 1 [file antioxidants-13-00757-s001.zip › antioxidants-3066162-supplementary.pdf]

uL3 Regulates Redox Metabolism and Ferroptosis Sensitivity of p53-Deleted Colorectal Cancer Cells

Chiara Brignola 1,†, Annalisa Pecoraro 1,†, Camilla Danisi 1, Nunzia Iaccarino 1, Anna Di Porzio 1, Francesca Romano 1, Pietro Carotenuto 2,3, Giulia Russo 1 and Annapina Russo 1,*

1 Department of Pharmacy, University of Naples "Federico II", Via Domenico Montesano, 49, 80131 Naples, Italy; chiara.brignola@unina.it (C.B.); annalisa.pecoraro@unina.it (A.P.); camilla.danisi@unina.it (C.D.); nunzia.iaccarino@unina.it (N.I.); anna.diporzio@unina.it (A.D.P.); francesca.romano2@unina.it (F.R.); giulia.russo@unina.it (G.R.)

2 TIGEM, Telethon Institute of Genetics and Medicine, Via Campi Flegrei, 34, 80078 Naples, Italy;
p.carotenuto@tigem.it

3 Medical Genetics, Department of Translational Medical Science, University of Naples "Federico II",
Corso Umberto I, 40, 80138 Naples, Italy

* Correspondence: annapina.russo@unina.it

† These authors contributed equally to this work.

a **b**

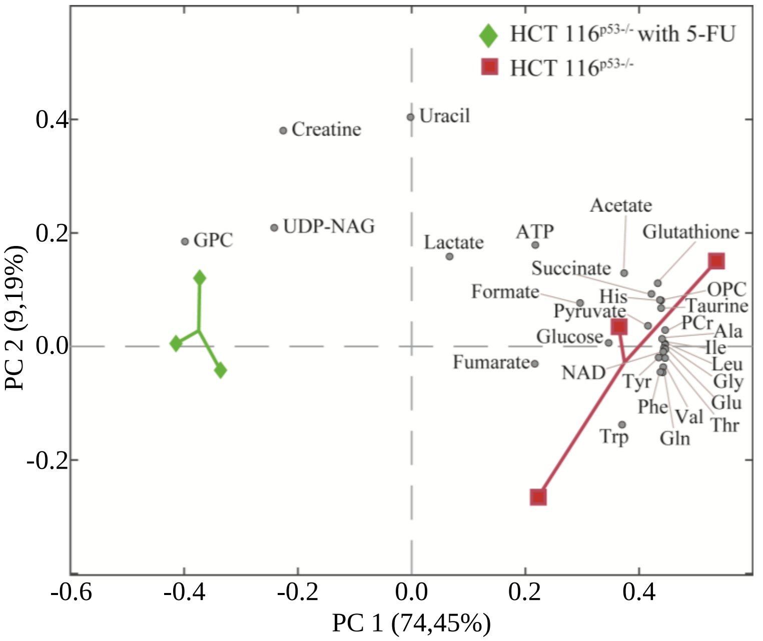

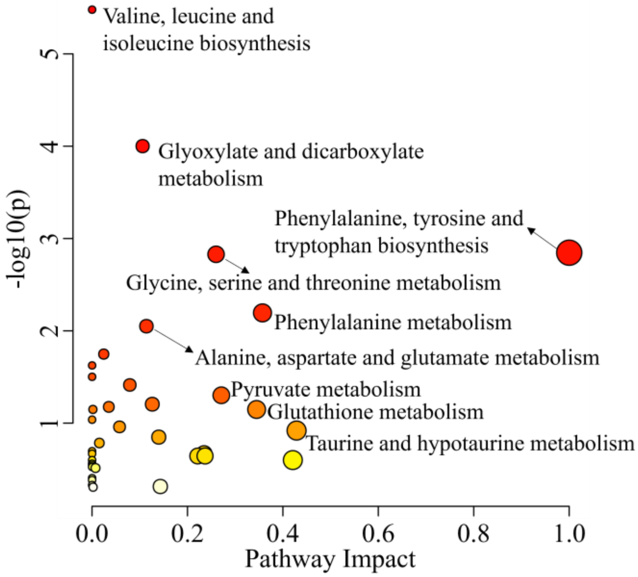

Figure S1. Effect of 5-FU treatment on metabolite profiles of HCT 116$^{p53-/-}$ cells. (**a**) Biplot (scores plot combined with loadings plot) of PCA model performed on the NMR-based metabolomic analyses of untreated HCT 116$^{p53-/-}$ cells vs. 5-FU-treated HCT 116$^{p53-/-}$ cells. (**b**) Relative pathway analyses (NAD, Nicotinamide adenine dinucleotide; CrP, Creatine Phosphate; UDP-NAG, Uridine diphosphate N-acetylglucosamine; OPC, O-phosphocholine; GPC, Glycerophosphocholine).

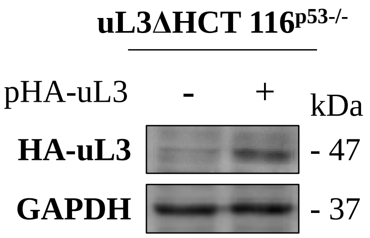

Figure S2. HA-uL3 expression levels in uL3ΔHCT 116$^{p53-/-}$ cells transfected with pHA-uL3 by WB. Cells were transiently transfected with pHA-uL3. 24 h later, protein extracts from the samples were analyzed by WB with antibodies against HA and GAPDH as loading control. Full-length blots are presented in Figure S6.

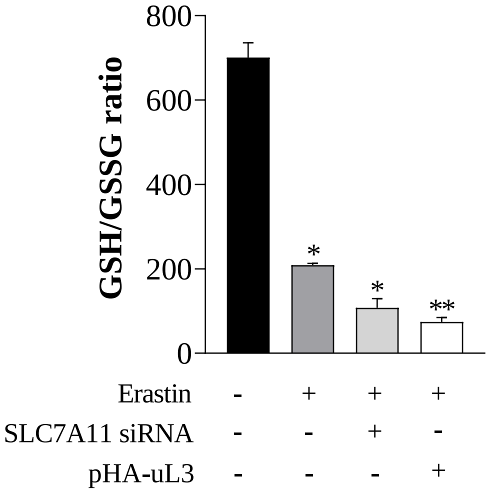

Figure S3. Evaluation of GSH/GSSG ratio. uL3ΔHCT 116$^{p53-/-}$ cells were transfected or not with SLC7A11 siRNA or pHA-uL3 and then, treated with erastin (10 µM). 24 h later, GSH was measured and GSH/GSSG ratio was calculated accordingly. **p* < 0.05, ***p* < 0.01 vs. control cells.

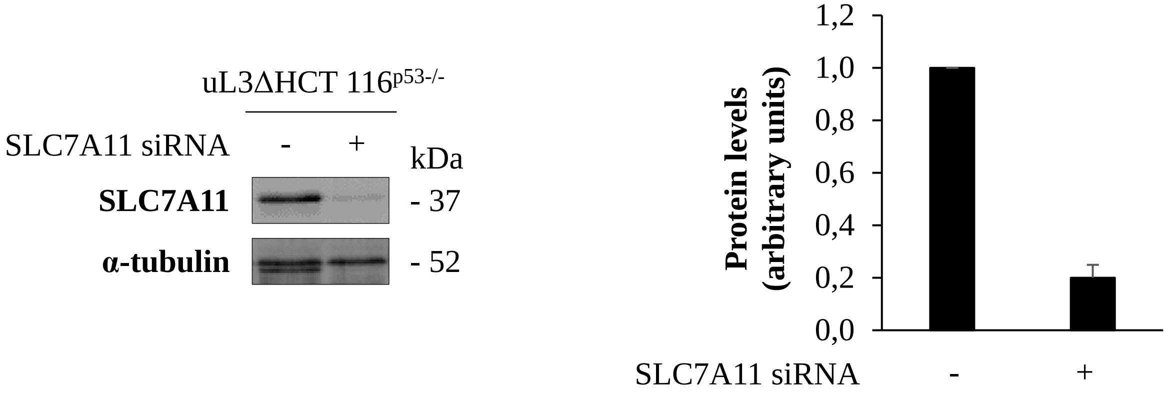

Figure S4. SLC7A11 expression levels in uL3ΔHCT 116$^{p53-/-}$ cells transfected with SLC7A11 siRNA. uL3ΔHCT 116$^{p53-/-}$ cells were transiently transfected SLC7A11 siRNA. 24 h later, protein extracts from amples were analyzed by WB with antibodies against SLC7A11 and α-tubulin as loading control. Full-length blots are presented in Figure S7.

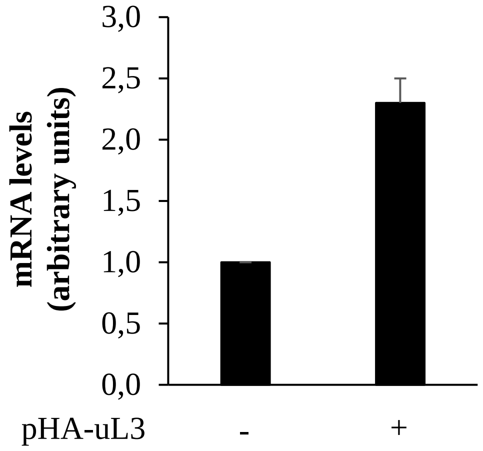

Figure S5. HA-uL3 expression levels in uL3ΔHCT 116$^{p53-/-}$ cells transfected with pHA-uL3 by RT-qPCR. Cells were transiently transfected with pHA-uL3. 12 h later, total RNA from samples were analyzed by RT-qPCR.

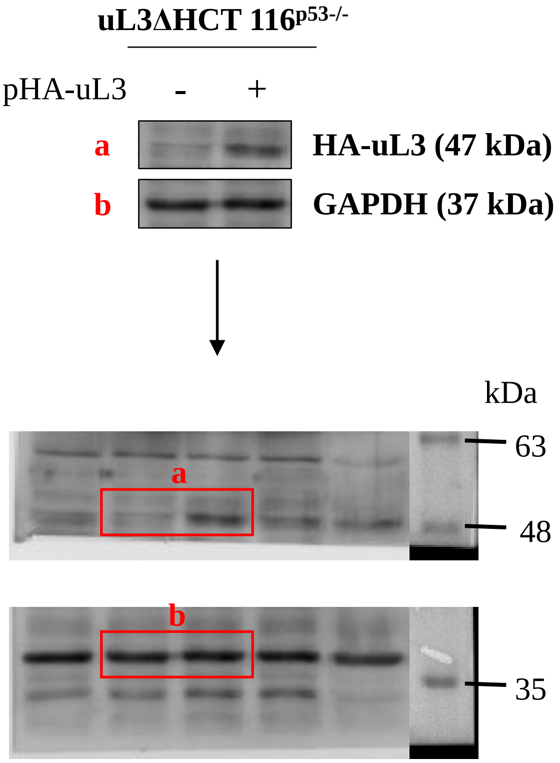

Figure S6. Full-length blots of Figure S2.

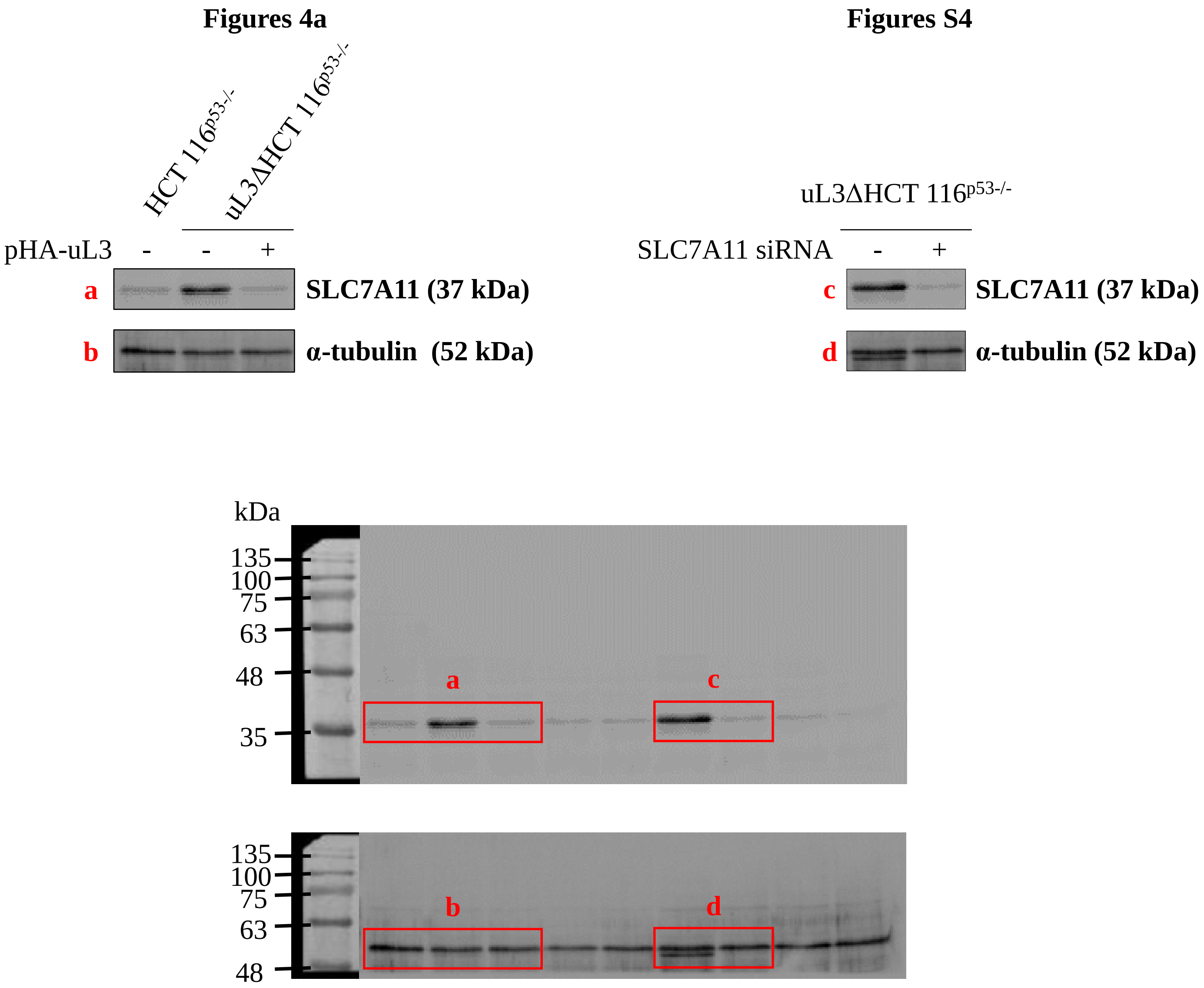

Figure S7. Full-length blots of Figures 4a and S4.

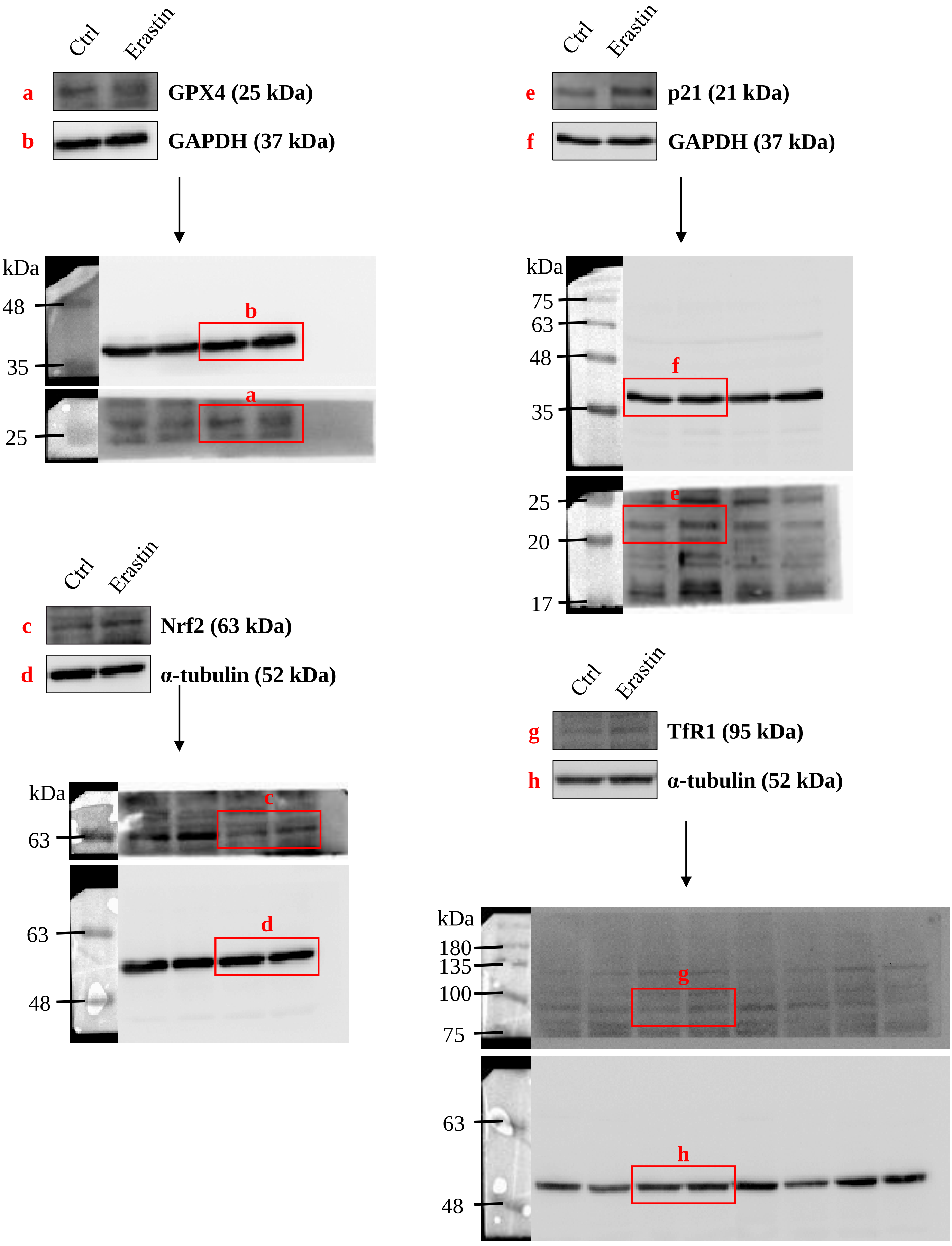

Figure S8. Full-length blots of Figure 6b.
